# Estimation of mosquito-borne and sexual transmission of Zika virus in Australia: Risks to blood transfusion safety

**DOI:** 10.1371/journal.pntd.0008438

**Published:** 2020-07-14

**Authors:** Elvina Viennet, Francesca D. Frentiu, Craig R. Williams, Gina Mincham, Cassie C. Jansen, Brian L. Montgomery, Robert L. P. Flower, Helen M. Faddy

**Affiliations:** 1 Research and Development, Australian Red Cross Lifeblood, Kelvin Grove, Queensland, Australia; 2 Institute for Health and Biomedical Innovation, School of Biomedical Sciences, Queensland University of Technology, Queensland, Australia; 3 Australian Centre for Precision Health, University of South Australia, Adelaide, South Australia, Australia; 4 Communicable Diseases Branch, Queensland Department of Health, Herston, Queensland, Australia; 5 Metro South Public Health Unit, Metro South Hospital and Health Service, Brisbane, Queensland, Australia; 6 School of Health and Sport Sciences, University of the Sunshine Coast, Queensland, Australia; London School of Hygiene & Tropical Medicine, UNITED KINGDOM

## Abstract

**Background:**

Since 2015, Zika virus (ZIKV) outbreaks have occurred in the Americas and the Pacific involving mosquito-borne and sexual transmission. ZIKV has also emerged as a risk to global blood transfusion safety. *Aedes aegypti*, a mosquito well established in north and some parts of central and southern Queensland, Australia, transmits ZIKV. *Aedes albopictus*, another potential ZIKV vector, is a threat to mainland Australia. Since these conditions create the potential for local transmission in Australia and a possible uncertainty in the effectiveness of blood donor risk-mitigation programs, we investigated the possible impact of mosquito-borne and sexual transmission of ZIKV in Australia on local blood transfusion safety.

**Methodology/Principal findings:**

We estimated ‘best-’ and ‘worst-’ case scenarios of monthly reproduction number (*R*_*0*_) for both transmission pathways of ZIKV from 1996–2015 in 11 urban or regional population centres, by varying epidemiological and entomological estimates. We then estimated the attack rate and subsequent number of infectious people to quantify the ZIKV transfusion-transmission risk using the European Up-Front Risk Assessment Tool. For all scenarios and with both vector species *R*_*0*_ was lower than one for ZIKV transmission. However, a higher risk of a sustained outbreak was estimated for Cairns, Rockhampton, Thursday Island, and theoretically in Darwin during the warmest months of the year. The yearly estimation of the risk of transmitting ZIKV infection by blood transfusion remained low through the study period for all locations, with the highest potential risk estimated in Darwin.

**Conclusions/Significance:**

Given the increasing demand for plasma products in Australia, the current strategy of restricting donors returning from infectious disease outbreak regions to source plasma collection provides a simple and effective risk management approach. However, if local transmission was suspected in the main urban centres of Australia, potentially facilitated by the geographic range expansion of *Ae*. *aegypti* or *Ae*. *albopictus*, this mitigation strategy would need urgent review.

## Author summary

What if Zika virus (ZIKV) had been introduced in Australia from 1996–2015 in urban centres with established mosquito vectors? Would Australian blood transfusion safety be compromised due to risks associated with ZIKV? In the event of ZIKV introduction into Australia during the period 1996–2015, our estimation shows that local transmission arising from an imported case, would have been possible in most of the selected urban centres, albeit very low. However, ZIKV transmission remained a potential threat in Cairns, Rockhampton, and Thursday Island during the warmest months. Potential geographic range expansion of both *Ae*. *albopictus* and *Ae*. *aegypti* is a key threat to the Australian mainland. We found that sexual transmission would have an important role in the estimation of *R*_*0*_ if only the *Ae*. *albopictus* population were present and not *Ae*. *aegypti*. Had both vectors been established in Australia during the period investigated and a ZIKV introduction occurred, the associated risk to blood transfusion safety would have been low. We identified regions of Australia where ZIKV transmission presents a potential, however low, concern for blood supply safety. Our study informs decision making on blood transfusion safety, public health and mosquito control policies.

## Introduction

Transmission of arboviruses via blood transfusion has been reported for dengue viruses (DENVs) and West Nile virus (WNV) [1], and suspected for other arboviruses. In Australia, outbreaks of DENVs and Ross River virus (RRV) threaten blood transfusion safety [2]. Zika virus (ZIKV), closely related to DENVs and WNV, is an emerging arbovirus with potential for transfusion-transmission [3–8]. ZIKV may be detected in serum or plasma for 1–2 weeks after infection and persists for longer periods of time in whole blood, red blood cells, semen, and urine [9–11]. Therefore, ZIKV presents yet another challenge to blood transfusion providers and to public health generally, as transfusion recipients could develop ZIKV-related disease following transfusion.

ZIKV belongs to the *Flavivirus* genus (family *Flaviridae*), along with DENVs and WNV, and is primarily transmitted to humans through the bite of an infected female *Aedes aegypti* or *Aedes albopictus* mosquito. Both species are principal vectors of DENVs and chikungunya virus (CHIKV). The former species is established in north Queensland and present in some towns in central and southern Queensland [12–14]. The latter species is not yet established in mainland Australia but is present on many Torres Strait islands [15] and is detected occasionally at Australia’s international air and seaports by the Department of Agriculture and Water Resources [16]. In addition to vector-borne transmission, sexual contact,[17] perinatal transmission,[18, 19] and blood transfusion [4, 5] are alternative transmission pathways for ZIKV. Symptomatic infection with ZIKV is usually characterized by mild, self-limiting febrile illness with low-grade fever, arthralgia, myalgia, headache and conjunctivitis [20], although as many as 80% of cases may be asymptomatic [50–80%][20, 21]. When acquired during pregnancy, ZIKV can cause miscarriage or neurological birth defects (e.g. microcephaly) [22]. In addition, ZIKV may also cause Guillain-Barré syndrome [23].

First isolated in Uganda in 1947, ZIKV was relatively unknown until an outbreak in Yap, Federated States of Micronesia, in 2007 and then in French Polynesia in 2013–2014. Outbreaks of ZIKV occurred throughout the Americas and the Pacific in 2015–2016. Local transmission has been reported in, at least, 87 countries and from 2015 to 2^nd^ July 2019 [24], and more than 33 million travel-associated cases of ZIKV have been reported from 2015 to week 4 2019 [25]. Vector competence of *Ae*. *aegypti* and *Ae*. *albopictus* mosquito populations for ZIKV varies substantially depending on the origin of the virus strain and mosquito population [26–34]. The vector competence of Australian *Ae*. *aegypti* has been established experimentally for African, Cambodian, Western Pacific and Brazilian virus strains [27, 35–37]. Hugo, Stassen *et al*. [35] recently showed that Australian *Ae*. *aegypti* can transmit a Brazilian epidemic ZIKV strain with more efficiency than *Ae*. *albopictus*. More than 140 cases of confirmed or probable ZIKV infection have been reported in travelers to Australia since 2012 as of 23 March 2019,[38–40] with the highest number of notifications reported in the areas of north Queensland where *Ae*. *aegypti* is present and abundant [41]. Areas of north-eastern Australia could sustain local transmission of ZIKV [41, 42] in wild-type *Ae*. *aegypti*, although the recent and expanding implementation of *Wolbachia*-based control strategies in the region may reduce future risk of potential transmission in locations where *Wolbachia*-infected *Ae*. *aegypti* persist in high relative densities [43, 44].

Australia has not yet reported local transmission of ZIKV. However, due to i) the close proximity to, and frequent air traffic with, the many neighboring countries endemic for ZIKV, ii) a history of imported cases acquired overseas, iii) the current favourable conditions for local transmission (suitable climate, presence of competent vectors) and, iv) a potential high rate of asymptomatic infection, transmission is plausible, and could pose a risk to public health. Furthermore, the potential for local outbreaks of ZIKV may extend to other regions of Australia, as the predicted risk of geographic range expansion of both *Ae*. *aegypti* and *Ae*. *albopictus* is high [15, 45].

While the risk to transfusion safety is currently small, a potential outbreak of ZIKV would threaten blood supply safety in vulnerable locations [42]. Minimization strategies and quantification of this risk are therefore warranted, should local transmission occur. Indeed, the Australian Red Cross Lifeblood (Lifeblood, formerly Blood Service) has several risk minimization strategies to ensure the risk of transfusion-transmitted ZIKV is minimal [46]. For example, blood donors must have i) satisfactorily completed a confidential interview and health assessment that includes a questionnaire on past and present medical conditions [47]; ii) satisfied minimum physiological criteria; iii) declared any high-risk behavior, practices and circumstances that prevents them from donating blood; iv) been instructed to contact Lifeblood with any information related to their health that could affect the suitability of their donation [48].

Accurate estimations of the risk of transfusion-transmitted ZIKV in Australia are essential for monitoring the safety of the blood supply and evaluating the effectiveness of the donor questionnaire and identifying the potential need for new screening tests. In this study, we hypothesized that if ZIKV were to be imported into a region with an established vector population during a period of suitable environmental conditions, transmission would be possible, and the blood transfusion supply would be potentially at risk. Although previous estimates of the epidemic potential by Viennet *et al*. [49] and Watson-Brown *et al*. [42] show that areas of north-eastern Australia could sustain local transmission of ZIKV, we now incorporate both vectors and explicitly account for sexual transmission. Based on previous work, we aimed to: i) calculate the basic reproduction numbers for ZIKV via mosquito-borne (*R*_*hv*_) and sexual (*R*_*hh*_) transmission in Australian Urban Centres and Localities (UCLs); ii) estimate contribution of ZIKV sexual transmission to *R*_*0*_; iii) estimate the number of people infected during potential local transmission events; and iv) assess the associated risk to blood transfusion safety. Estimating the risk of ZIKV transfusion-transmission is an important part of risk assessments and informs decisions regarding when and which risk mitigation strategies should be implemented.

## Methods

### Study area and data sources

Eleven UCLs in Australia were selected for modelling: Adelaide, Hobart, and Melbourne, which have never had established *Ae*. *aegypti* populations; Brisbane, Darwin, Perth, and Sydney, which previously had established *Ae*. *aegypti* populations but for which there is no recent evidence of permanent *Ae*. *aegypti* populations; and Cairns, Rockhampton, Thursday Island (Torres Strait) and Townsville, which have established *Ae*. *aegypti* populations (and also have experienced local dengue virus activity) [13, 14, 45]. The density of human populations were obtained from the Australian Bureau of Statistics [50] from the 1996, 2001, 2006 and 2011 census data with linear interpolation for inter-census years. *Aedes albopictus* is known only to be established in the Torres Strait, Australia [15, 51]. We obtained the mean maximum and minimum temperatures from the Australian Bureau of Meteorology and calculated the average temperature for each month from January 1996 –December 2015 [52]. Finally, we used the container-inhabiting mosquito simulation (CIMSiM) model, which is driven by daily meteorological observations, mosquito food availability, availability of various breeding sites (containers) and human demographic data to estimate the density of *Ae*. *aegypti* and *Ae*. *albopictus* host-seeking females [53], that we defined below as mosquito population density. CIMSiM has been previously validated for its ability to simulate *Ae*. *aegypti* productivity in Australia [54].

### Estimation of the basic reproduction number

*R*_*hv*_ was used to estimate the epidemic potential of a vector-pathogen combination [55], while *R*_*hh*_ was used to estimate the epidemic potential of sexual transmission. Given that there has been no reported local transmission of ZIKV in Australia, estimation of the basic reproduction number for vector-borne transmission of ZIKV was based on Gao *et al*. [56] and given by:

$$R_{hv}=\sqrt{(\frac{b^{2}{\times \beta }_{m}\times \beta_{h}\times \rho \times \frac{M_{L}}{H_{L}}\times c\times \theta }{\gamma_{H}\times \mu_{v}}+\frac{b^{2}{\times \beta }_{m}\times \beta_{h}\times \frac{M_{L}}{H_{L}}\times c\times \theta }{\gamma_{H1}\times \mu_{v}})\times \frac{\gamma_{v}}{\gamma_{v}+\mu_{v}}},$$ (1)

The estimation of the basic reproduction number for sexual transmission (*R*_*hh*_) of ZIKV was based on Gao *et al*. [56] and given by:

$$R_{hh}=\frac{K\beta \theta }{\gamma_{H}}+\frac{\theta \beta }{\gamma_{H1}}+\frac{\tau \theta \beta }{\gamma_{H2}},$$ (2)

Finally, the basic reproduction number *R*_*0*_, defined as the number of secondary infections produced per day from a single primary infection introduced into an immunologically naïve population [57] is given by the following equation [56]:

$$R_{0}=\frac{R_{hh}+\sqrt{R_{hh}^{2}+4R_{hv}^{2}}}{2},$$ (3)

We estimated the monthly *R*_*hv*_, *R*_*hh*_ and *R*_*0*_ through the study period. *R*_*0*_ is estimated for the ‘best-’ and ‘worst-’ case scenarios, based on the mean value of *R*_*hh*_, and the values of *R*_*hv*_ (‘best-’ and ‘worst-’ case scenarios). In general, an epidemic could occur in a susceptible population when *R*_*0*_ > 1. *R*_*0*_ can be calculated using notified cases from a known epidemic (direct method) or by assuming the introduction of a single infective case of virus and modelling viral transmission based on known parameters (indirect method). We also estimated the average *R*_*hv*_ of the warmest six months (November to April) under the ‘worst-’ and ‘best-’ case scenarios (defined below).

Estimation was performed using the software R [58] (version 3.4.1). Several assumptions were made to perform the analyses. ZIKV was assumed to be introduced into an immunologically naïve population. We also hypothesized that: i) either an *Ae*. *aegypti* population or an *Ae*. *albopictus* population is the only vector present in each UCL (i.e. not both at once); ii) the mosquito population is modelled accurately by CIMSiM and that all *Ae*. *aegypti* are wild-type (not infected with *Wolbachia*); iii) the human population undergoes linear growth throughout study period and ignores births and deaths; iv) vector control is present and consistent between UCLs; v) only symptomatic infections are considered infectious; vi) the protective effect of herd immunity is not considered. All parameter descriptions are summarized in S1 Table (S1 Table), with rationale for each explained in Watson-Brown *et al*. [42].

### Scenarios considered

The estimation was done using the upper and bounds of two key parameters that demonstrate the effect of environmental factors on transmission intensity [i.e. efficiency of vector control (*c*), and extrinsic incubation period (EIP = **1**/***γ***_***v***_)], and with different probabilities of human-to-vector infections per bite (*β*_*m*_) and of vector-to-human transmission per bite (*β*_*h*_). We used a set of *β*_*m*_ and *β*_*h*_, observed by Hall-Mendelin *et al*. [37], Duchemin *et al*. [27] and Hugo, Stassen *et al*. [35] for *Ae*. *aegypti*, and that of Duchemin *et al*. [27] and Hugo, Stassen et al [35] for *Ae*. *albopictus*. The combinations of upper and lower bounds of the 95% confidence interval (CI) around *c* and duration of *τ* together with the most relevant set of *β*_*m*_ and *β*_*h*_ for our study were utilized to describe theoretical ‘best-’ and ‘worst-’ case scenarios. The ‘relevance’ was defined by the homogeneity of experiments between the two mosquito species, the origin of the mosquito population and the origin of the ZIKV strain (ideally, tied to epidemics). The ‘best-case’ scenario used a longer EIP (high *τ*) and high efficiency of vector control (low *c*), while the ‘worst-case’ scenario assumed a short EIP (low *τ*) and low efficiency of vector control (high *c*).

### Uncertainty and sensitivity analyses

To quantify the impact of the variation of each parameter on the outcome variable in the *R*_*hv*_ (1), and *R*_*hh*_ Eq (2) for *Ae*. *aegypti* and *Ae*. *albopictus*, we combined uncertainty analysis through the Monte Carlo simulations (MCS) approach with the sensitivity analysis over other parameter variations through robust Partial rank correlation coefficient (PRCC) method [56, 59, 60]. We used the MCS method to generate 100,000 samples uniformly distributed in the range of parameter values (S1 Table) and calculated the corresponding uncertainty on the reproduction number via mosquito-borne and sexual transmission. A matrix was generated with 100,000 rows representing the number of simulations and the number of columns corresponding to the number of varied parameters (*AvgT*,*β*_*m*_,*β*_*h*_,*ρ*,*θ*,*γ*_*H*_,*γ*_*H*1_,*γ*_*v*_,*M*_*L*_,*H*_*L*_,*c*,*μ*_*v*_,*b*) and ($K,\beta ,\tau ,\theta ,\frac{1}{\partial_{H}},\frac{1}{\gamma_{H1}},\frac{1}{\gamma_{H2}}$) involved in *R*_*hv*_ and *R*_*hh*_ calculations, respectively. Finally, to identify the key factors that determine the magnitude of the basic reproduction number, we computed the PRCCs between *R*_*hv*_ and each of its parameters, as well as *R*_*hh*_ and each of its parameters.

### Attack rate

The attack rate, referred to below as *AR*, is one of the most important quantities that describes the severity of an epidemic and expresses the fraction of individuals who might become infected. Under a susceptible-infected-recovered transmission model, we generated location-specific projections of *AR* based on its theoretical relationship with the basic reproduction number, *R*_*0*_ [61, 62]:

AR=1−S

With *S*, the proportion of remaining susceptible after the epidemic has burned out is given by:

$$S=e^{-R_{0}(1-S)}$$

The *ARs* have been calculated for the ‘best-‘and ‘worst-’ case scenarios of *R*_*0*_ for ZIKV by UCLs over the study period. Then, to obtain the mean number of potential infected people by year and UCLs, we multiplied their given mean *AR* by the corresponding yearly human population.

### Risk for blood safety

Finally, we utilized the European Up-Front Risk Assessment Tool (EUFRAT- version 2.2.31, http://eufrattool.ecdc.europa.eu/) with estimated cumulative infections to assess the transfusion-transmission risk from predicted potentially infected blood donors. EUFRAT has a web-based interface and was commissioned by the European Centre for Disease Prevention and Control (ECDC) to quantify the transmission risk through blood transfusion during outbreaks of emerging infectious diseases (EIDs) [63–67]. The conceptual basis of the EUFRAT is further described in Kiely *et al*. (2017) [68]. Several assumptions were made to perform the analyses with EUFRAT: (i) blood donors had the same risk as any other individual; (ii) there was no effect of infection on donation behaviour; (iii) infections were evenly distributed over the interval considered; iv) the duration of infectivity was constant and fixed at 14 days; v) blood components (red blood cells, platelets, and plasma products) from viraemic blood donors transmit infection with 100% efficiency. EUFRAT does allow the user to enter a value for the proportion of the population that is immune, if that is known. If the proportion of the population that is immune is not known, the usual conservative assumption is to assume 0% immunity (i.e. 100% transmissibility), which is the case in this study.

Data regarding fresh blood components collected and number of blood donations issued were available for all UCLs (except Thursday Island) at the Statistical Area level 3 (SA3) from 2009 onwards. SA3 are geographical areas built from whole Statistical Areas Level 2 (SA2), themselves built from whole Statistical Areas Level 1 (SA1) [69, 70]. An Urban Centre is a cluster of contiguous SA1s with an aggregate population exceeding 1,000 persons contained within SA1s that are 'of urban character' [71]. There is considerable crossover with the SA3 level, which enabled a reasonable aggregation of SA3s to UCLs. Consequently, we estimated the yearly transfusion-transmission risk from 2009 to 2015 by UCLs.

Based on an asymptomatic rate of ZIKV infection ranging from 50 to 80% [20, 21], we fixed the proportion of undetected cases at 65%. As explained above, the number of infections reported was estimated by year and UCLs using the *AR*. The population size represented the number of individuals in the outbreak-affected region (UCL here) and estimated previously. The blood component production data from 2009 to 2015 were obtained from Lifeblood databases. The other parameters were taken from Coghlan *et al*. [66]. We then estimated the risk of infected components being released by dividing the total number of products obtained from individual donations per year N with the estimated number of infected components released in year N. Given the severe clinical consequences to an infected foetus, the large proportion of asymptomatic cases, and the detection of ZIKV RNA in asymptomatic blood donors, we purposely did not adopt a numerical risk threshold as a trigger for additional risk mitigation for ZIKV, as this would have been too subjective, but rather adopted levels of risk (very low, low, medium, high) as a trigger for additional risk mitigation for ZIKV.

## Results

### Predicted distribution of *Ae*. *aegypti* and *Ae*. *albopictus*

The distribution of *Ae*. *aegypti* and *Ae*. *albopictus* simulated via CIMSiM are illustrated in the S1 and S2 Figs, respectively. As expected, the density of *Ae*. *aegypti* and *Ae*. *albopictus* populations after introduction declined over time in Adelaide, Hobart and Melbourne. In Perth, while the density of the population declined for the former, it slightly increased over time for the latter. As for the other UCLs, the density of the *Ae*. *aegypti* population did not change much over time, while *Ae*. *albopictus* density decreased in Darwin and increased in Sydney over time. For both species, the correlation between the density of host-seeking females (mosquito population density) was slightly above average (R = 0.6, p < 2.2e-16) (S1 and S2 Figs).

### Basic reproduction number

The *R*_*hv*_ ‘best-’ and ‘worst-’ case scenarios were calculated monthly from January 1996 to December 2015 in each of the 11 UCLs for *Ae*. *aegypti* and *Ae*. *albopictus* based on a set of different estimates of *β*_*m*_ and *β*_*h*_. As per Watson-Brown *et al*. [42], the average *R*_*hv*_, *R*_*hh*_, and *R*_*0*_ of the warmer six months (November to April) under the ‘worst-’ and ‘best-’ scenarios provide a single estimate for each modelled location (Table 1). This provides a higher overall estimate than if all months were included. For both *Ae*. *aegypti* and *Ae*. *albopictus*, *R*_*hv*_ and the resulting *R*_*0*_ were below 1, which means that established outbreaks would have been eliminated with any interventions to maintain *R*_*0*_ < 1. Cairns had the highest overall *R*_*0*_ and therefore the greatest risk of an outbreak (e.g. *R*_*0*_ *=* 0.89 (95% CI: 0.80–0.99) with *Ae*. *aegypti*; *R*_*0*_ *=* 0.66 (95% CI: 0.54–0.78) with *Ae*. *albopictus*). Then, for *Ae*. *aegypti*, Cairns was followed by Rockhampton, Darwin, Thursday Island, Townsville and Brisbane, while for *Ae*. *albopictus*, Cairns was followed by Darwin, Thursday Island, Rockhampton, Townsville and Brisbane.

**Table 1:** Global reproduction number *R*_*0*_ with the reproduction number for mosquito-borne (*Aedes aegypti* and *Aedes albopictus*) and sexual transmission over the study period (November-April 1996–2015) in UCLs of Australia.

				Urban Centre and Localities										
Mosquito species	Parameter based on		Scenario	Adelaide	Brisbane	Cairns	Darwin	Hobart	Melbourne	Perth	Rockhampton	Sydney	Thursday Island	Townsville
***Aedes*** ***aegypti***	**Mean ZIKV *R***_***hv***_ **(95% CI)**	**Hall-Mendelin et al. (2016)**	**‘Best-case’**	0.00	0.17 (0.13–0.22)	0.28 (0.25–0.31)	0.23 (0.15–0.31)	0.00	0.00	0.00	0.24 (0.18–0.30)	0.00	0.21 (0.13–0.29)	0.17 (0.11–0.22)
			**‘Worst-case’**	0.00	0.33 (0.24–0.41)	0.52 (0.46–0.59)	0.43 (0.29–0.58)	0.00	0.00	0.00	0.45 (0.33–0.56)	0.00	0.39 (0.25–0.54)	0.31 (0.21–0.41)
		**Duchemin et al. (2017)**	**BC**	0.00	0.38 (0.29–0.48)	0.61 (0.54–0.69)	0.51 (0.34–0.68)	0.00	0.00	0.00	0.52 (0.39–0.65)	0.00	0.46 (0.29–0.63)	0.36 (0.24–0.49)
			**WC**	0.00	0.71 (0.53–0.89)	1.14 (1.01–1.28)	0.94 (0.63–1.26)	0.00	0.00	0.00	0.97 (0.73–1.22)	0.00	0.86 (0.55–1.18)	0.68 (0.45–0.90)
		**Hugo, Stassen et al. (2019)**	**BC**	0.00	0.28 (0.21–0.35)	0.45 (0.39–0.50)	0.37 (0.24–0.49)	0.00	0.00	0.00	0.38 (0.28–0.48)	0.00	0.34 (0.21–0.46)	0.26 (0.18–0.35)
			**WC**	0.00	0.52 (0.39–0.65)	0.83 (0.73–0.93)	0.69 (0.46–0.92)	0.00	0.00	0.00	0.71 (0.53–0.89)	0.00	0.63 (0.40–0.85)	0.49 (0.33–0.66)
	**Mean ZIKV *R***_***hh***_ **[range]**			0.12 [0.00–0.25]	0.12 [0.00–0.25]	0.12 [0.00–0.25]	0.12 [0.00–0.25]	0.12 0.00–0.25]	0.12 [0.00–0.25]	0.12 [0.00–0.25]	0.12 [0.00–0.25]	0.12 [0.00–0.25]	0.12 [0.00–0.25]	0.12 [0.00–0.25]
	**Mean ZIKV *R***_***0***_ **(95% CI)**	**Hall-Mendelin et al. (2016)**	**BC**	0.12	0.24 (0.20–0.28)	0.35 (0.31–0.38)	0.30 (0.22–0.38)	0.12	0.12	0.12	0.31 (0.25–0.36)	0.12	0.28 (0.21–0.35)	0.24 (0.18–0.29)
			**WC**	0.12	0.39 (0.31–0.47)	0.59 (0.53–0.65)	0.50 (0.35–0.64)	0.12	0.12	0.12	0.51 (0.40–0.62)	0.12	0.46 (0.32–0.60)	0.38 (0.28–0.48))
		**Duchemin et al. (2017)**	**BC**	0.12	0.45 (0.35–0.54))	0.68 (0.60–0.75)	0.57 (0.40–0.74)	0.12	0.12	0.12	0.59 (0.46–0.72)	0.12	0.53 (0.36–0.69)	0.43 (0.31–0.55)
			**WC**	0.12	0.77 (0.60–0.95)	1.2 (1.07–1.34)	1.00 (0.69–1.32)	0.12	0.12	0.12	1.03 (0.79–1.28)	0.12	0.92 (0.61–1.24)	0.74 (0.52–0.97))
		**Hugo, Stassen et al. (2019)**	**BC**	0.12	0.34 (0.28–0.41)	0.51 (0.46–0.56)	0.43 (0.31–0.56)	0.12	0.12	0.12	0.44 (0.35–0.54)	0.12	0.40 (0.28–0.52)	0.33 (0.25–0.42)
			**WC**	0.12	0.58 (0.45–0.71)	0.89 (0.80–0.99)	0.75 (0.52–0.98)	0.12	0.12	0.12	0.77 (0.59–0.95)	0.12	0.69 (0.46–0.92)	0.56 (0.40–0.72)
***Aedes*** ***albopictus***	**Mean ZIKV *R***_***hv***_ **(95% CI)**	**Duchemin et al. (2017)**	**BC**	0.00	0.04 (0.0–0.07)	0.10 (0.08–0.12)	0.07 (0.04–0.11)	0.00	0.00	0.00	0.03 (0.02–0.11)	0.01 (0.00–0.03))	0.07 (0.04–0.10)	0.05 (0.02–0.07)
			**WC**	0.00	0.26 (0.05–0.47)	0.60 (0.48–0.72)	0.45 (0.25–0.65)	0.00	0.00	0.00	0.40 (0.14–0.66)	0.09 (0.01–0.20)	0.43 (0.24–0.62)	0.31 (0.17–0.46)
		**Hugo, Stassen et al. (2019)**	**BC**	0.00	0.01 (0.00–0.03)	0.04 (0.03–0.04)	0.03 (0.01–0.04)	0.00	0.00	0.00	0.02 (0.00–0.04)	0.01 (0.00–0.01)	0.02 (0.01–0.04)	0.02 (0.01–0.03)
			**WC**	0.00	0.10 (0.02–0.19)	0.24 (0.19–0.29)	0.18 (0.10–0.26)	0.00	0.00	0.00	0.16 (0.05–0.26)	0.03 (0.00–0.08)	0.17 (0.09–0.25)	2.58 (2.44–2.72)
	**Mean ZIKV *R***_***hh***_ **[range]**			0.12 [0.00–0.25]	0.12 [0.00–0.25]	0.12 [0.00–0.25]	0.12 [0.00–0.25]	0.12 [0.00–0.25]	0.12 [0.00–0.25]	0.12 [0.00–0.25]	0.12 [0.00–0.25]	0.12 [0.00–0.25]	0.12 [0.00–0.25]	0.12 [0.00–0.25]
	**Mean ZIKV *R***_***0***_ **(95% CI)**	**Duchemin et al. (2017)**	**BC**	0.12	0.14 (0.12–0.16)	0.17 (0.16–0.19)	0.15 (0.13–0.18)	0.12	0.12	0.12	0.15 (0.12–0.18)	0.12 (0.11–0.13)	0.15 (0.13–0.18)	0.14 (0.12–0.15)
			**WC**	0.12	0.34 (0.15–0.53)	0.66 (0.54–0.78)	0.51 (0.31–0.72)	0.12	0.12	0.12	0.47 (0.22–0.72)	0.19 (0.10–0.28)	0.49 (0.30–0.68)	0.38 (0.24–0.52)
		**Hugo, Stassen et al. (2019)**	**BC**	0.12	0.14 (0.12–0.16)	0.17 (0.16–0.19)	0.15 (0.13–0.18)	0.12	0.12	0.12	0.15 (0.12–0.18)	0.12 (0.11–0.13)	0.15 (0.13–0.18)	0.14 (0.12–0.15)
			**WC**	0.12	0.34 (0.15–0.53)	0.66 (0.54–0.78)	0.51 (0.31–0.72)	0.12	0.12	0.12	0.47 (0.22–0.72)	0.19 (0.10–0.28)	0.49 (0.30–0.68)	0.38 (0.24–0.52)

*BC =* ‘Best-Case’*; WC =* ‘Worst-Case’*; R*_*hv*_ = basic reproduction number for mosquito-borne transmission; *R*_*hh*_ = basic reproduction number for sexual transmission; *R*_*0*_ = basic reproduction number; *CI* = confidence interval; ZIKV = Zika virus

*R_*hv*_ estimates < 0.001 were entered as 0.00. UCL = Urban Centre and Locality (see S1 Table)

**Fig 1:**
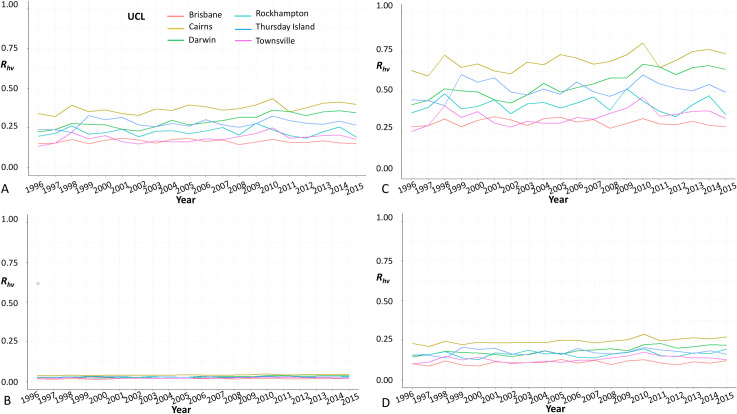
Yearly *R*_*hv*_ estimates based on ‘best-’ and ‘worst-’case scenarios from January 1996 to December 2015 in each of the 11 UCLs. (A) Best-case scenario with *Aedes aegypti*; (B) Worst-case scenario with *Aedes aegypti;* (C) Best-case scenario with *Aedes albopictus;* (D) Worst-case scenario with *Aedes albopictus*.

The set of *β*_*m*_ and *β*_*h*_ parameters given by Hall-Mendelin *et al*. [37] provides the lowest *R*_*hv*_ values, while the set of *β*_*m*_ and *β*_*h*_ parameters given by Duchemin *et al*. [27] provides the highest values. For consistency, we present the results only based on the set of parameters published by Hugo, Stassen *et al*. (2019), providing an intermediate set of *β*_*m*_ and *β*_*h*_ parameters. Fig 1 presents locations where *R*_*hv*_ >0 throughout the entire year (i.e. Brisbane, Cairns, Darwin, Rockhampton, Thursday Island, Townsville) (Fig 1). The locations were the same when either species of mosquito were considered. For both species, *R*_*hv*_ was highest in Cairns, followed by Darwin, Thursday Island, Rockhampton, Townsville and finally Brisbane.

**Fig 2:**
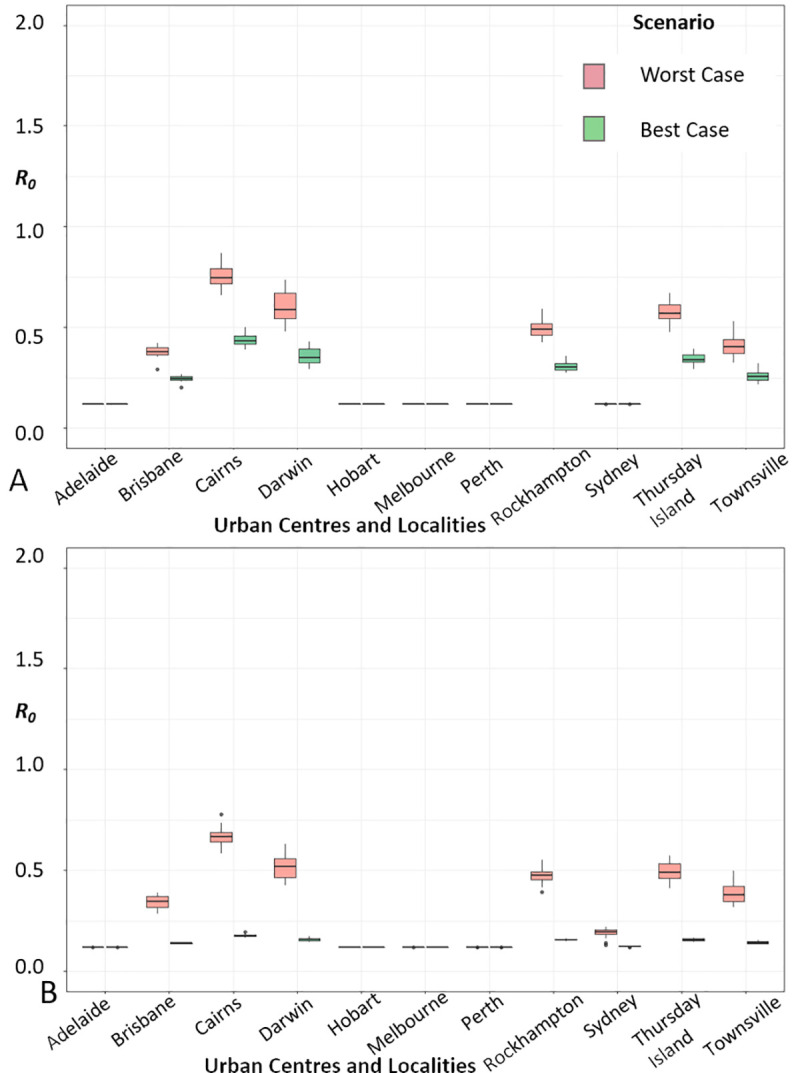
Global *R*_*0*_ throughout the study period for the UCLs studied. *R*_*0*_ ‘worst-’ and ‘best-’case scenarios (A) with *Aedes aegypti;* (B) with *Aedes albopictus*.

**Fig 3:**
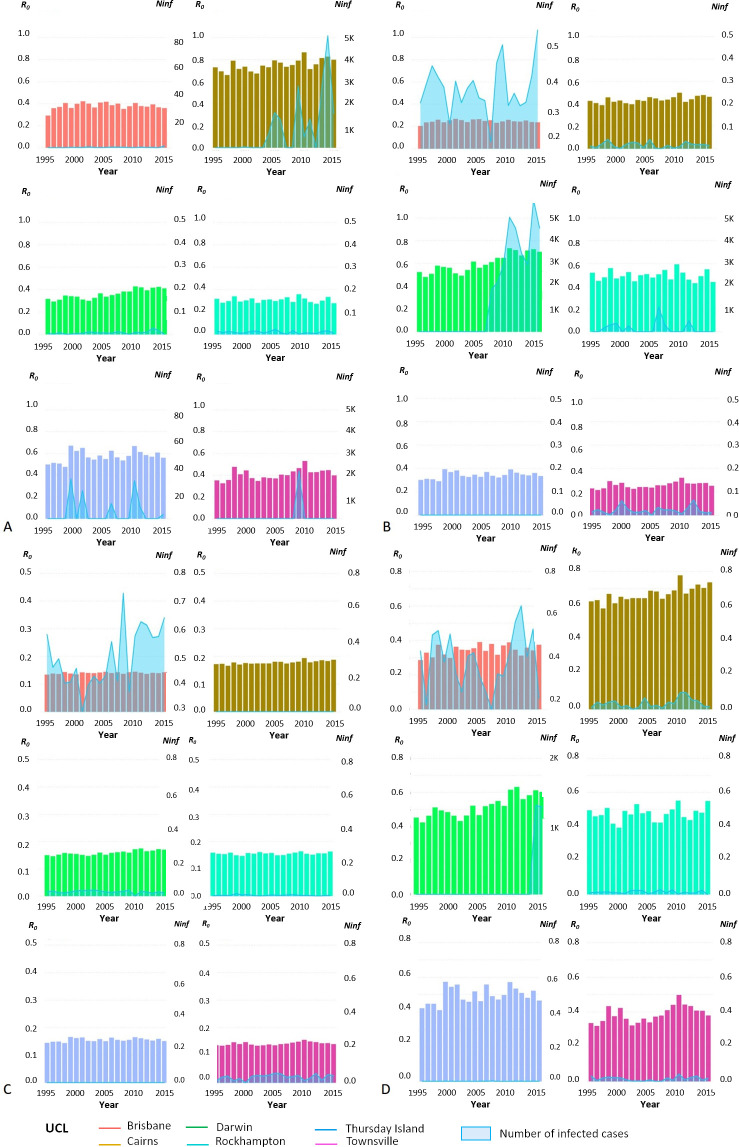
Yearly *R*_*0*_ and estimated number of infected people with ZIKV throughout the study period for the UCLs studied. (A) ‘best-‘case scenario with *Aedes aegypti;* (B) ‘worst-‘case scenario with *Aedes aegypti;* (C) ‘best-‘case scenario with *Aedes albopictus;* (D) ‘worst-‘case scenario with *Aedes albopictus*.

The reproduction number due to sexual transmission and the basic reproduction number *R*_*0*_ were estimated based on Eqs (2) and (3) in Methods, respectively. Despite a few exceptions through the study period, the global mean *R*_*0*_ (Fig 2; Fig 3; S3 Fig) was higher in Cairns followed by Darwin, Thursday Island, Rockhampton, Townsville and Brisbane.

### Uncertainty and sensitivity analyses

For both mosquito species, we plotted the median and interquartile range (IQR) of the estimates *R*_*0*_ (‘worst-’ and ‘best-’case scenarios), and *R*_*hv*_ (‘worst-‘and ‘best-’case scenarios) (S4 Fig). Quartiles are best used for population distributions that are irregularly shaped or asymmetric because they are insensitive to outliers and preserve information about the centre and spread of the data [72]. However, as an indication, we also provided the mean and standard deviation. The difference between the median of the ‘worst-’ and the median of the ‘best-’ case scenario parameters is larger for *Ae*. *albopictus* than for *Ae*. *aegypti*. The length of the boxes is the IQR and measures the spread of the data (25 and 75 percentile). *R*_*0*_ and *R*_*hv*_ ‘worst-case’ scenario have greater variability than *R*_*0*_ and *R*_*hv*_ ‘best- case’ scenario. For *Ae*. *albopictus*, the boxplots representing the ‘best-case’ scenarios are comparatively short, which suggests that overall, the values describing *R*_*0*_ and *R*_*hv*_ are similar. The notch displays the 95% confidence interval around the median, while the Tukey-style whiskers represent the reasonable extremes of the data, extended to a maximum of 1.5*IQR beyond the box. For both mosquito species, the minimum and maximum values of *R*_*0*_ and *R*_*hv*_ ‘worst-’ and ‘best-’case scenarios do not exceed these extremes.

**Fig 4:**
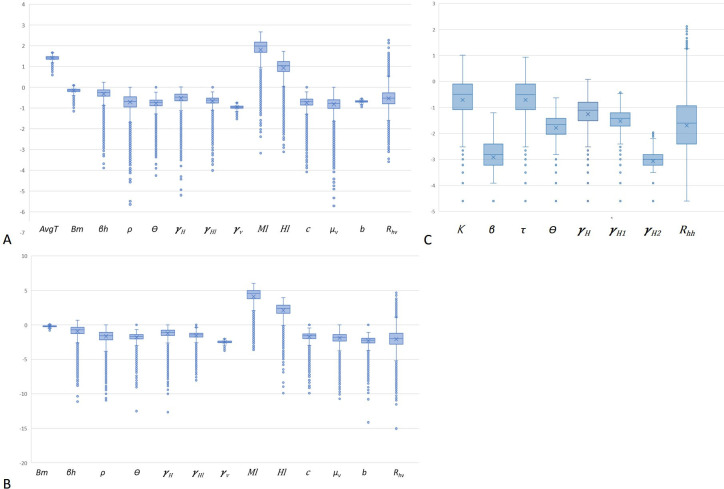
Uncertainty analyses. Box plot of the log of the basic reproduction number reproduction number for mosquito-borne transmission *R*_*hv*_ (A) *Aedes aegyti*; (B) *Aedes albopictus* and reproduction number for sexual transmission *R*_*hh*_ (C) *Ae*. *aegypti* and *Ae*. *albopictus R*_*hv*_: Reproduction number for mosquito-borne transmission; *AvgT*: average temperature; *H*_*L*_: Human population density; *M*_*L*:_ Mosquito population density; *ρ*: Relative human-to-mosquito transmission probability of exposed humans to symptomatically infected humans (per day); *γ*_*H*1_: inverse of the duration of acute phase; *γ*_*H*_: Inverse of the intrinsic incubation period in humans; *θ*: Proportion of symptomatic infections; *γ*_*v*_: inverse of the duration of extrinsic incubation; *β*_*h*_: probability of vector to human transmission per bite; *β*_*m*_: probability of human to vector transmission per bite; *μ*_*v*_: Inverse of the mosquito lifespan; *c*: vector control rate; b: average daily vector biting rate; *R*_*hh*_: Reproduction number for sexual transmission; *K*: Relative human-to-human transmissibility of exposed humans to symptomatic humans; beta: *β*: Transmission rate from symptomatically infected humans to susceptible humans; tau: *τ*: Relative human-to-human transmissibility of convalescent to symptomatic humans; theta: *θ*: Proportion of symptomatic infections; *γ*_*H*_: Inverse of the intrinsic incubation period in humans; *γ*_*H*1_: Inverse of the duration of acute phase; *γ*_*H*2_: Inverse of the duration of convalescent phase.

For both vector species, variability was mainly observed in the relative human-to-mosquito transmission probability of exposed humans to symptomatically infected humans (*ρ*), the mosquito density (*M*_*L*_), the human population density (*M*_*L*_) and the inverse of the mosquito lifespan (*μ*_*v*_)_used to estimate the overall *R*_*hv*_. Variability was also principally observed in the relative human-to-human transmissibility of exposed humans to symptomatic humans (*K*) and the inverse of the duration of extrinsic incubation period (***γ***_***v***_) used in *R*_*hh*_ (Fig 4).

To identify the key parameters that affect the *R*_*hv*_ for both species and *R*_*hh*_, we performed a sensitivity analysis with 100,000 random samples uniformly distributed in the range of the parameters from S1 Table and using the Monte Carlo simulations approach. For *Ae*. *aegypti*, *R*_*hv*_ was most sensitive to mosquito population and human population densities. For *Ae*. *albopictus*, *R*_*hv*_ was most sensitive to human population density and the biting rate. Finally, *R*_*hh*_ was most sensitive to the duration of acute phase, the intrinsic incubation period and the transmission rate from symptomatically infected humans to susceptible humans (S5 Fig).

**Fig 5:**
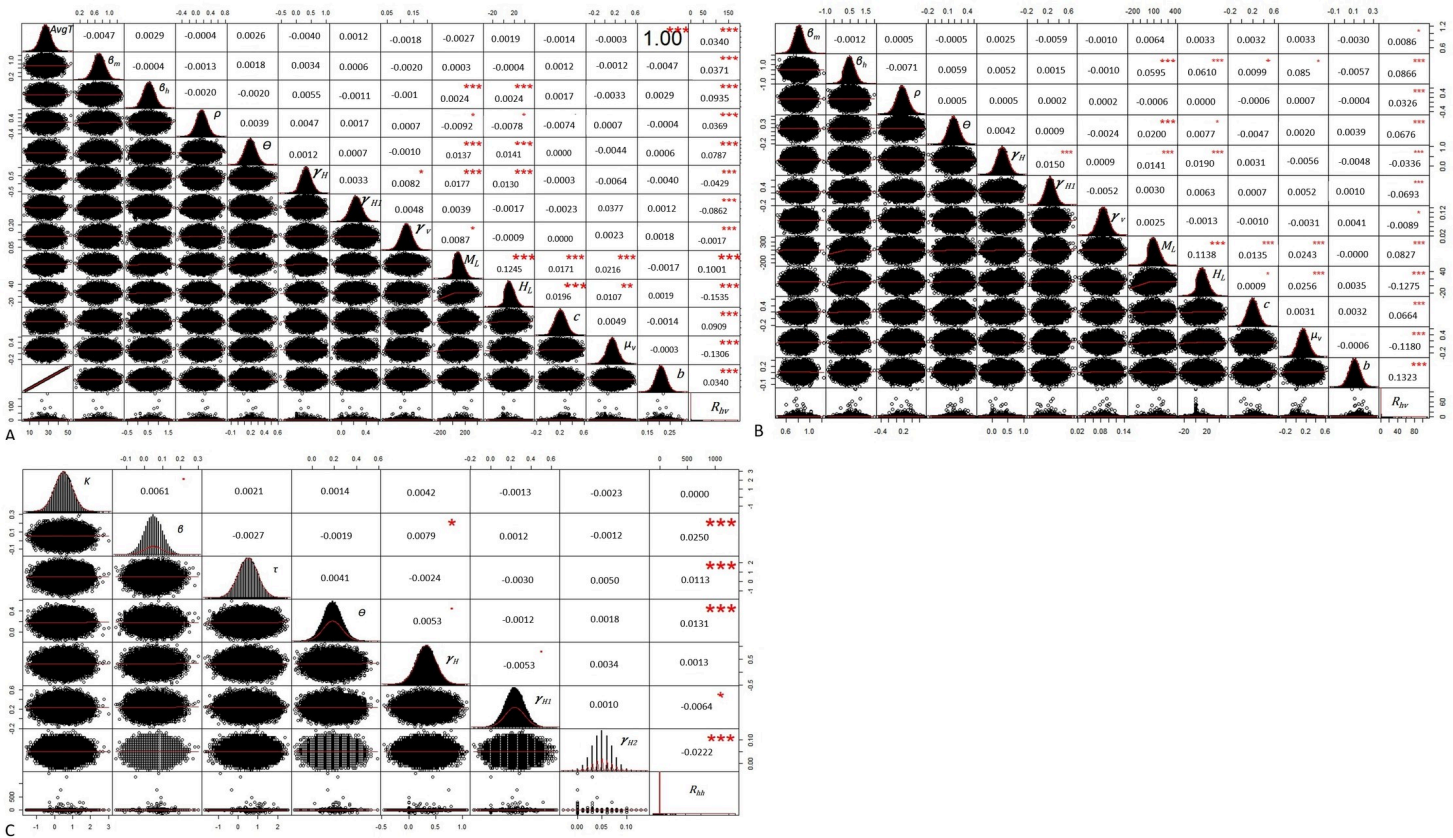
Sensitivity analyses. Correlation matrix for all pairs of the observed variables, to be read from the diagonal. The distribution of each variable is shown on the left bottom of the diagonal; the bivariate scatter plots with a fitted line are displayed right top of the diagonal; the upper panel gives the corresponding Pearson correlation coefficient with the significance level as *. In the upper panel, the correlation coefficient is tested against the null hypothesis and the resulting p-value is shown at the levels of 0.1 ('), 0.05 (*), 0.01 (**), and 0.001 (***). (A) *R*_*hv*_ with *Aedes aegypti*; (B) *R*_*hv*_ with *Aedes albopictus;* (C) *R*_*hh*_ with *Ae*. *aegypti* and *Ae*. *Albopictus R*_*hv*_: Reproduction number for mosquito-borne transmission; *AvgT*: average temperature; *H*_*L*_: Human population density; *M*_*L*:_ Mosquito population density; *ρ*: Relative human-to-mosquito transmission probability of exposed humans to symptomatically infected humans (per day); *γ*_*H*1_: inverse of the duration of acute phase; *γ*_*H*_: Inverse of the intrinsic incubation period in humans; *θ*: Proportion of symptomatic infections; *γ*_*v*_: inverse of the duration of extrinsic incubation; *β*_*h*_: probability of vector to human transmission per bite; *β*_*m*_: probability of human to vector transmission per bite; *μ*_*v*_: Inverse of the mosquito lifespan; *c*: vector control rate; b: average daily vector biting rate; *R*_*hh*_: Reproduction number for sexual transmission; *K*: Relative human-to-human transmissibility of exposed humans to symptomatic humans; beta: *β*: Transmission rate from symptomatically infected humans to susceptible humans; tau: *τ*: Relative human-to-human transmissibility of convalescent to symptomatic humans; theta: *θ*: Proportion of symptomatic infections; *γ*_*H*_: Inverse of the intrinsic incubation period in humans; *γ*_*H*1_: Inverse of the duration of acute phase; *γ*_*H*2_: Inverse of the duration of convalescent phase.

Sensitivity analyses for *Ae*. *aegypti* showed that all parameters except the intrinsic incubation period in humans, the duration of acute phase, the duration of extrinsic incubation period, the mosquito lifespan and the human population density had a significant and low positive impact on *R*_*hv*_ outcomes (Fig 5A). Also as expected, the average temperature was significantly and highly correlated to the biting rate (R^2^ = 1). The mosquito population and human population densities had a significant and low positive impact on the probability of vector to human transmission per bite, the proportion of symptomatic infections and the intrinsic incubation period in humans. For *Ae*. *albopictus*, the mosquito population density, probability of vector to human transmission per bite, vector control rate, proportion of symptomatic infections, the relative human-to-mosquito transmission probability of exposed humans to symptomatically infected humans and average daily vector biting rate had a significant and low positive impact on *R*_*hv*_ outcomes (Fig 5B). For both species, the transmission rate from symptomatically infected humans to susceptible humans, the relative human-to-human transmissibility of convalescent to symptomatic humans, the proportion of symptomatic infections had a significant and positive impact on *R*_*hh*_ outcomes while the duration of convalescent phase. had a negative correlation with *R*_*hh*_ (Fig 5C).

### Contribution by sexual transmission in *R*_*0*_

The relative contribution of transmission by sexual activity to overall transmission is presented in Table 2, together with the attack rate from November to April 1996–2015 in the 11 Australian UCLs. In Brisbane, Cairns, Darwin, Rockhampton, Thursday Island and Townsville, sexual transmission contributed less than 63.0% in *R*_*0*_ (‘best-case’ scenario) and less than 35.0% in *R*_*0*_ (‘worst-case’ scenario) for *Aedes aegypti*, while for *Aedes albopictus*, sexual transmission represented the major contribution of ZIKV transmission (above 98%).

**Table 2:** Percentages of contribution by ZIKV sexual transmission in the basic reproduction number (*Aedes aegypti* and *Aedes albopictus*), and attack rate over the study period (November-April 1996–2015) in UCLs of Australia.

				Urban Centre and Localities										
Mosquito species	Paramater based on		Scenario	Adelaide	Brisbane	Cairns	Darwin	Hobart	Melbourne	Perth	Rockhampton	Sydney	Thursday Island	Townsville
***Aedes*** ***aegypti***	***Rp* (%)**	**Hall-Mendelin et al. (2016)**	**‘Best-Case’**	100.0	79.0	59.5	68.2	100.0	100.0	100.0	66.9	100.0	72.1	80.5
			**‘Worst-Case’**	100.0	52.4	30.0	38.5	100.0	100.0	100.0	37.2	100.0	43.0	54.7
		**Duchemin et al. (2017)**	**BC**	100.0	44.5	23.8	31.4	100.0	100.0	100.0	30.1	100.0	35.5	46.8
			**WC**	100.0	19.0	8.3	11.7	100.0	100.0	100.0	11.2	100.0	13.8	20.4
		**Hugo, Stassen et al. (2019)**	**BC**	100.0	60.1	37.0	46.2	100.0	100.0	100.0	44.7	100.0	50.8	62.3
			**WC**	100.0	30.5	14.6	34.5	100.0	100.0	100.0	19.1	100.0	23.1	32.5
	***AR (%) (95% CI)***	**Hall-Mendelin et al. (2016)**	**BC**	0.0	0.0	0.0	0.0	0.0	0.0	0.0	0.0	0.0	0.0	0.0
			**WC**	0.0	0.0	0.0	0.0	0.0	0.0	0.0	0.0	0.0	0.0	0.0
		**Duchemin et al. (2017)**	**BC**	0.0	0.0	0.0	0.0	0.0	0.0	0.0	0.0	0.0	0.0	0.0
			**WC**	0.0	0.3 (0.0–2.0)	30.3 (14.6–46.0)	17.8 (0.00–39.9)	0.0	0.0	0.0	17.4 (0.00–34.9)	0.0	15.5 (0.0–34.2)	3.5 (0.0–13.7)
		**Hugo, Stassen et al. (2019)**	**BC**	0.0	0.0	0.0	0.0	0.0	0.0	0.0	0.0	0.0	0.0	0.0
			**WC**	0.0	0.0	1.2 (0.0–4.8)	3.0 (0.0–11.2)	0.0	0.0	0.0	0.4 (0.0–2.7)	0.0	0.3 (0.0–2.3)	0.1 (0.0–1.6)
***Aedes*** ***albopictus***	***Rp* (%)**	**Duchemin et al. (2017)**	**BC**	100.0	98.3	92.1	95.4	100.0	100.0	99.9	96.3	99.7	95.8	97.6
			**WC**	100.0	62.8	24.6	36.7	100.0	100.0	99.9	42.3	92.5	39.2	53.9
		**Hugo, Stassen et al. (2019)**	**BC**	100.0	99.7	98.6	99.2	100.0	100.0	99.9	99.3	99.9	99.3	99.6
			**WC**	100.0	99.7	98.6	99.2	100.0	100.0	99.9	99.3	99.9	99.3	99.6
	***AR (%) (95% CI)***	**Duchemin et al. (2017)**	**BC**	0.0	0.0	0.0	0.0	0.0	0.0	0.0	0.0	0.0	0.0	0.0
			**WC**	0.0	0.0	0.0	0.0	0.0	0.0	0.0	0.0	0.0	0.0	0.0
		**Hugo, Stassen et al. (2019)**	**BC**	0.0	0.0	0.0	0.0	0.0	0.0	0.0	0.0	0.0	0.0	0.0
			**WC**	0.0	0.0	0.0	0.0	0.0	0.0	0.0	0.0	0.0	0.0	0.0

*BC =* ‘Best-Case’*; WC =* ‘Worst-Case’; *AR =* attack rate*; R*_*p*_ = *R*_*hh*_ /(*R*_*hh*_ + *R*^*2*^_*hv*_)*100, percentages of contribution by sexual transmission in the basic reproduction number, defined by Gao et al.^44^; CI = confidence interval

### Attack rate

The attack rate was null for both species, except with *Ae*. *aegypti* for Cairns, Darwin, Rockhampton, Thursday Island, and Townsville ranging from 0.1 to 3 for the ‘worst-‘case scenario (Table 2). The biggest AR for the ‘worst-‘case scenario was in Darwin followed by Cairns and Rockhampton. Our results show, to a certain extent, that reducing the mosquito population together with a longer EIP would only partly prevent transmission (or contain an outbreak).

### Potential number of infected people and risk for blood safety

**Fig 6:**
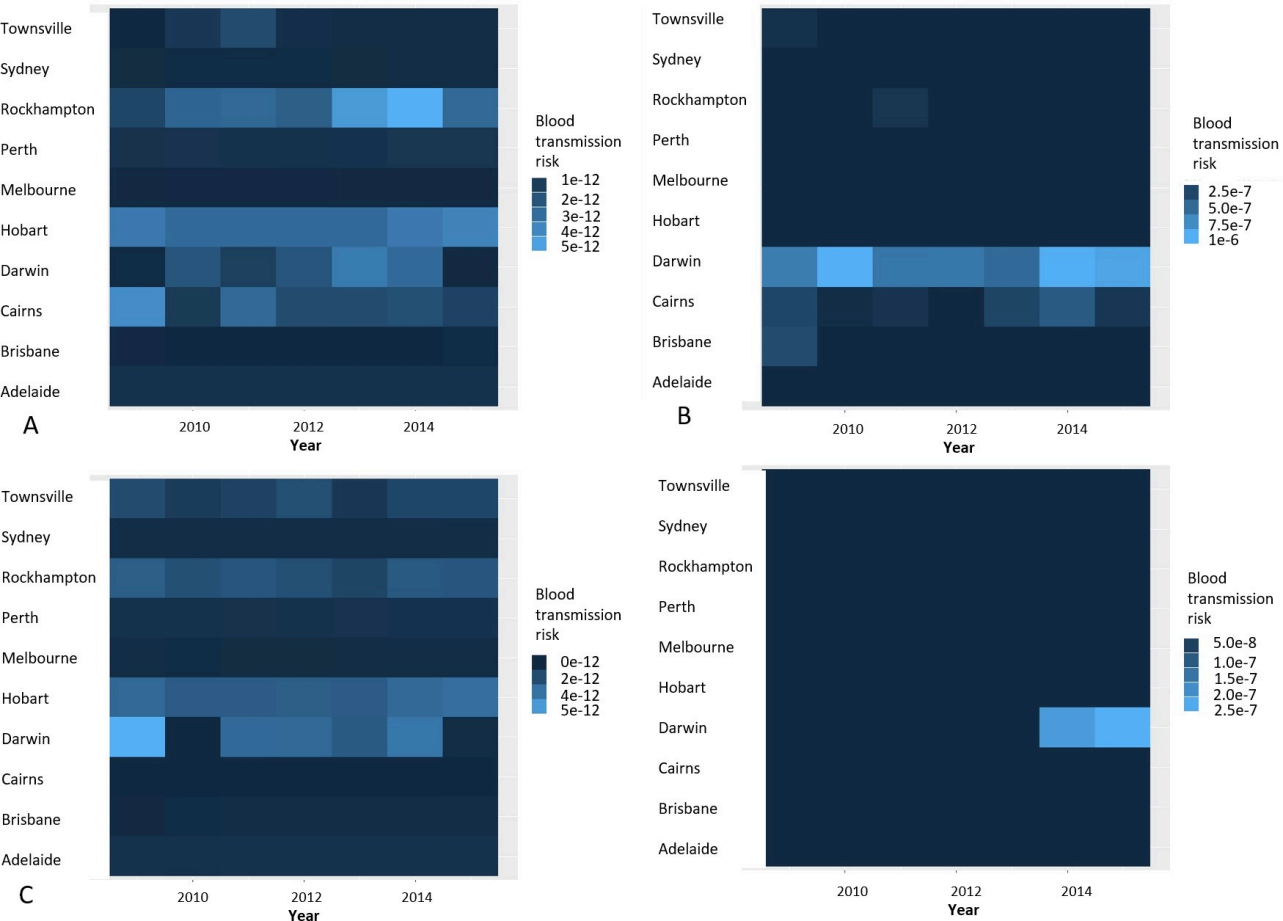
Blood transmission risk. Heat map of the blood transmission risk by Urban Centres and Localities throughout from 2009–2015. (A) ‘best-‘case scenario with *Aedes aegypti;* (B) ‘worst-‘case scenario with *Ae*. *aegypti;* (C) ‘best-‘case scenario with *Aedes albopictus;* (D) ‘worst-‘case scenario with *Ae*. *Albopictus*.

Once the yearly attack rate was calculated, we estimated the potential number of infected people (Fig 3) and the potential risk to blood transfusion safety by UCL (apart from Thursday Island) and year for the ‘best-’ and ‘worst-’ case scenarios based on Hugo, Stassen *et al*.’s parameters. Of the mean yearly number of blood donations across the ten UCLs, the origins of 54.8% were concentrated from the more populated areas of Victoria and New South Wales (the cities of Sydney and Melbourne), where there was no epidemic potential for ZIKV by *Ae*. *aegypti*. Areas with epidemic potential for ZIKV affected 24.7% of the mean yearly number of blood donations from the ten UCLs, with the greatest average number of donations residing in Brisbane (171,727) followed by Townsville (23,600), Cairns (9,273), Rockhampton (6,904) and Darwin (5,685). Cairns, Rockhampton and Townsville represented only 4.5% of the annual mean number of blood donations in the studied UCLs. Thursday Island did not have any recorded donors. Regarding the potential number of infected products and subsequent risk for blood safety, there was no substantial risk identified with the best-case scenario, while the risk was extremely low with the worst-case scenario. The highest estimate of predicted likelihood of infection in blood components were from donations in Darwin [one in 992,681 with *Ae*. *aegypti*, one in 3,836,000 with *Ae*. *albopictus*], Cairns [one in 2,474,000 with *Ae*. *aegypti;* null with *Ae*. *albopictus*], Rockhampton [one in 9,657,000 with *Ae*. *aegypti;* null with *Ae*. *albopictus*] UCLs (Fig 6).

## Discussion

Following the emergence of ZIKV in the Pacific and Latin America and given the serious sequelae accompanying congenital ZIKV exposure, extensive research has focused on better understanding and characterizing the consequences of ZIKV infection and transmission. Moreover, the possibility of transmitting infectious organisms via blood products and plasma derivatives is a major public health concern. This study fills a gap in our knowledge on the potential risk associated with ZIKV for Australian blood transfusion safety. We attempted to assess i) whether an epidemic could occur in main urban centres in Australia, following ZIKV introduction in areas with suitable conditions, and ii) how the safety of the blood supply would be impacted.

Hall-Mendelin *et al*.[37] found a relatively low probability of infection (57%) and transmission (27%) of ZIKV while two later studies found a higher rate of ZIKV infection (83% [27], 70% [35]) and transmission (87% [27], 55% [35]) in Australian populations of *Ae*. *aegypti* lacking *Wolbachia* infections, suggesting that local *Ae*. *aegypti* may be a more competent vector of ZIKV in Australia than previously thought [27]. Our study extends that of Hall-Mendelin et al [37] as we considered in our estimation three sets of probability of human-to-vector infection per bite (*β*_*m*_) and probability of vector-to-human transmission per bite for the local populations of *Ae*. *aegypti* and *Ae*. *albopictus* to account for uncertainty.

The predicted potential distribution of *Ae*. *aegypti* encompasses Cairns, Rockhampton, Thursday Island and Townsville, where the vector population is currently established, as well as Brisbane and Darwin, where *Ae*. *aegypti* used to be established. The predicted potential distribution of *Ae*. *albopictus* contains not only Thursday Island, where the species is established, but its presence is also predicted in Brisbane, Cairns, Darwin, Perth, Rockhampton, Sydney, and Townsville, which corroborate Kraemer *et al*. (2015)’s findings [73].

Kucharski *et al*. (2016) and Rahman *et al*. (2019) used a mathematical model to examine the 2013–14 outbreak in the six major archipelagos of French Polynesia. They found that ZIKV may exhibit similar dynamics to dengue virus with a *R*_*0*_ ranging from 2.6–4.8 [74], and 2.03–3.20 [75] and that reducing mosquito-to-human contact by at least 60% can reduce the peak prevalence by nearly 10% [75]. In the event of ZIKV introduction into Australia in the period 1996–2015, our study shows that from an imported infected case, the yearly epidemic potential would have been below one for all UCLs studied, though potentially really close to one in Cairns, Rockhampton, and Thursday Island during the warmer months where *Ae*. *aegypti* is established. The risk of an outbreak remains theoretically low in Darwin, where *Ae*. *aegypti* was established in the past, but for which there is currently no evidence of persisting populations. These results suggest a temperature suitability of Darwin for ZIKV transmission, should *Ae*. *aegypti* mosquitoes become established there again. Likewise, theoretically an epidemic of ZIKV would not be able to spread in these four UCLs, if only *Ae*. *albopictus* was established. However, due to its presence in the Torres Strait and frequent detections at international First Points of Entry, preventing its expansion onto the Australian mainland remains a key priority [27]. Our analyses also highlight a modest contribution of sexual transmission to the basic reproduction number when only *Ae*. *Aegypti* is considered, whereas a high contribution with only *Ae*. *albopictus*.

Uncertainty and sensitivity analyses offered a way to assess the adequacy of the *R*_*hv*_, and *R*_*hh*_ estimations and established which factors affect outputs. We showed that, to decrease the likelihood of ZIKV transmission through a mosquito bite, the priority should be to decrease the mosquito density, the probability of vector to human transmission, and the vector control rate. These priorities are reflected in the current strategies implemented in North Queensland to manage Dengue outbreak risk. Similarly, to decrease the likelihood of ZIKV transmission through sexual activities, the priority should be to decrease the transmission rate from symptomatically infected humans to susceptible humans, the relative human-to-human transmissibility of convalescent to symptomatic humans and, theoretically, the proportion of symptomatic infections. Accordingly, World Health Organization published guidance and recommendations, that are regularly updated as new evidence emerges, to provide advice on the prevention of sexual transmission of ZIKV [76].

In our previous study, we found that blood donations were predominantly distributed around the large urban centres of Sydney and Melbourne, which did not have epidemic potential for ZIKV by either mosquito species [42]. Local transmission of ZIKV in Cairns, Rockhampton or Townsville presented the highest risk to the blood supply, although these combined locations represent only 4.5% of the annual mean number of blood donations in Australia. Of the UCLs where outbreaks are presently possible due to the presence of *Ae*. *aegypti*, Cairns, and Rockhampton are the most at risk of having infected blood components obtained from individual donations with the ‘worst-‘case scenario (noting that Rockhampton is the only region without predominantly *Wolbachia*-infected strains of *Ae*. *aegypti* which may reduce transmission). Moreover, a risk of infected blood components obtained from individual donations would arise in Darwin, if *Ae*. *aegypti* was to re-establish. If *Ae*. *albopictus* was established, the possibility of having infected blood components obtained from individual donations would only be possible with the ‘worst-‘case scenario in Darwin UCL. By use of the most conservative estimates (‘worst-case’ scenario), the risk of collecting a viraemic donation could have been as high as one in 992,681 (in Darwin in 2010 with *Ae*. *aegypti*), and one in 4,448,512 (again in Darwin in 2014 with *Ae*. *albopictus*), which are still very low risks.

The proportion of symptomatic ZIKV infections [66, 77–80] ranges from 17–35% in Flamand *et al*. [79], to 20% in Lazear *et al*. [78] and Coghlan *et al*. [66], and 20–50% in Joguet *et al*. [80]. In order to avoid underestimating the risks for blood supply safety, we opted for an estimate of 20% symptomatic ZIKV infections. We also reasoned that a higher proportion of symptomatic acute infections would be identified and excluded through the blood donor questionnaire, which would further reduce the risk of collecting contaminated blood from asymptomatic donors.

This study has some limitations. First, our estimation is based on predicted mosquito population density rather than empirical data which was impossible to obtain due to a lack of consistent and sensitive mosquito survey, and rarely available due to typically heterogeneous distributions over highly focal geographic area exhibited by both these species. Second, we cannot strictly compare our estimates of *R*_*hv*_, and *R*_*0*_ to those published as the calculation depends on the model used and the geographical locations. In addition, we have not dissociated genders in the equation of sexual transmission, as the proportion of male to female transmission is higher than vice-versa [81]. Moreover, we have not considered human mobility, which is an important factor in arbovirus transmission. *R*_*hv*_, *R*_*hh*_ and *R*_*0*_ give a picture of the epidemic potential at time *t*, once an infected person is introduced, and therefore, we cannot indicate how the established outbreaks will behave (fade out, sustain or peak). The mean maximum mosquito lifespan (age of the oldest survivor) is difficult to observe under field conditions. Mousson et al (2010) estimated *Ae albopictus*’s lifespan, however we argue that the appropriate term in their study should be “life expectancy”, not “lifespan”. Therefore, we assumed the same lifespan used in Gao et al (2016) for both vectors. We integrated a range of vector control rates, which broadly pictured the effect of relevant mosquito control in Australia. Notably, as recently introduced *Wolbachia* infection in some Australian populations of *Ae*. *aegypti* would likely impact the ability of mosquitoes to transmit ZIKV [82], it would be beneficial to consider the impact of *Wolbachia* infection on transmission potential across relevant locations. One of the sources of parameter uncertainty in EUFRAT is the uncertainty around population incidence due to misdiagnosis, underreporting, lack of laboratory confirmation and proportion of asymptomatic cases. Nonetheless, misdiagnosis and underreporting issues that are common during ZIKV epidemics due to non-specific clinical presentation [83], do not affect our risk assessment because we are not estimating *R*_*0*_ with case notifications. While in the absence of empirical data, the risk estimates are valuable, the inherent uncertainties challenge the interpretation of their significance [84]. Finally, we simplified the infectious period of ZIKV to a single parameter based on a median 14 days from symptom onset till loss of viral RNA in serum [9]. It is thought that presymptomatic transmission may occur and others have attempted to include this parameter in modelling efforts [56]. The effect of not including presymptomatic transmission directly in our estimation is likely to be minimal because we used a long symptomatic infectious period compared with other models.

This study contributes to assessing the epidemic potential of local ZIKV transmission via mosquito-borne and sexual transmission, and provides, for the first time, an estimation of the risk posed by ZIKV for blood transfusion safety in Australia. Our methods and results differ from Watson-Brown *et al*. (2019) and highlight the difficulty in comparing different models. The salient point here is that each method can potentially produce a different estimate of *R*_*0*_. Therefore, using the basic reproduction number to predict an attack rate and ultimately, in our case, the risk for blood transfusion safety is dependent on the model employed. Nonetheless, estimation of *R*_*0*_ can potentially provide valuable insights during epidemics and guide public health interventions. We believe that the methodology employed in our study is easily reproducible in settings where epidemiological and entomological parameters are well estimated and could be used to allow a timely assessment of arboviruses, such as dengue, chikungunya and Zika viruses.

This work emphasizes the importance of ongoing vector surveillance and management programs to reduce the threat posed by *Ae*. *aegypti* and *Ae*. *albopictus* in areas at risk, and to prevent their establishment into new areas with theoretical epidemic potential. It also underscores the need to pursue investment in alternative risk–mitigation strategies, such as pathogen inactivation (PI) or nucleic acid amplification testing (NAT) for maintaining blood transfusion safety in the face of an infectious disease outbreak. PI refers to prevention of infectivity of a pathogen through chemical and/or physical removal processes (e.g. nanofiltration) to inactivate infectious disease agents (i.e. viruses, bacteria and parasites) from blood [85, 86].

To limit the risk of any ZIKV case in Australia and more importantly of foetal blood transfusion resulting in permanent disability, i) mosquito surveillance and vector control programs should be sustained and tailored to reduce the mosquito population density (*M*_*L*_) and the probability of vector to human transmission per bite (*β*_*h*_) with a view to understanding future impacts to regions likely to be targeted for release of *Wolbachia*-modified *Ae*. *aegypti*, and ii) Lifeblood’s travel deferrals policy, presently appropriate, should be maintained as it is. In any event of ZIKV outbreak, however, blood donors, who have visited the outbreak area, would be restricted to plasma for fractionation for 28 days post-exposure. Moreover, to mitigate infectious risk to blood safety, in event of infectious disease outbreak, Queensland Health Department notifies the Lifeblood by following an infectious disease outbreak protocol.

In conclusion, our findings comprise theoretical evidence that ZIKV did not present a large threat to Australia but could become one if transmitted by *Ae*. *aegypti* and/or *Ae*. *albopictus* in Cairns, Darwin, Rockhampton and Thursday Island UCLs during the warmer months of the year, provided all the environmental conditions suitable for transmission were met. Mosquito surveillance and vector control are integral to preventing the range expansion of both vectors, especially into those locations with theoretical epidemic potential (i.e. Darwin). While the acute threat from the 2015–2016 ZIKV epidemics may have subsided and the current level of imported cases in Australia is low, preparedness for local transmission of ZIKV remains important, as an outbreak could have a significant impact on health, tourism and the blood supply. Although risk estimates necessarily include a measure of uncertainty, our risk assessment nonetheless provides a dynamic estimate of ZIKV epidemic potential and risk level for blood transfusion safety in key urban centres and localities of Australia and has the potential to inform decision making relating to the timing of supplementary fresh component restriction measures. This approach may be useful and applicable for other countries (France, Italy, Japan, and United States of America) where there is no endemic transmission of ZIKV, but vector populations are present and sexually transmitted infections are reported.

## Supporting information

S1 Table Description of parameters used in *R*_*hv*_ *and R*_*hh*_ calculation. (DOCX)

S1 Fig Predicted distribution of *Aedes aegypti*. (A) Distribution from CIMSiM estimations through study period; (B) Analysis of correlation between the density of population and the Temperature (⁰C).
(TIF)

S2 Fig Predicted distribution of *Aedes albopictus*. (A) Distribution from CIMSiM estimations through study period; (B) Analysis of correlation between the density of population and the Temperature (⁰C).
(TIF)

S3 Fig Basic reproduction number for Zika virus. Heat map of *R*_*0*_ by Urban Centres and Localities throughout from 2009–2015. (A) ‘best-‘case scenario with *Aedes aegypti;* (B) ‘worst-‘case scenario with *Ae*. *aegypti;* (C) ‘best-‘case scenario with *Aedes albopictus;* (D) ‘worst-‘case scenario with *Ae*. *Albopictus* 1.Adelaide; 2. Brisbane; 3. Cairns; 4. Darwin; 5. Hobart; 6. Melbourne; 7. Perth; 8. Rockhampton; 9. Sydney; 10. Thursday Island; 11. Townsville.
(TIF)

S4 Fig Box plot representing the basic reproduction number for mosquito-borne transmission (*R*_*hv*_ ‘best-case’ and ‘worst-case’ scenario) and global mean basic reproduction number *R*_*0*_ in Urban Centres and Localities from 1996–2015. (A) Notched Box plots for *Aedes aegypti*; (B) Notched Box plots for *Aedes albopictus*. The box shows the interquartile range. The whiskers add 1.5 times the IQR to the 75 percentiles and subtract 1.5 times the IQR from the 25 percentiles. The line shows the median of the data. The notch displays the confidence interval around the median which is normally based on the median.
(TIF)

S5 Fig Partial Rank Correlation Coefficient (PRCC) of the reproduction number. (A) via mosquito transmission and *Aedes aegypti*; (B) via mosquito transmission and *Aedes albopictus;*(C) via sexual transmission.
(TIF)
