# Supplementary material for: Estimation of mosquito-borne and sexual transmission of Zika virus in Australia: Risks to blood transfusion safety
Source: PLoS Negl Trop Dis. 2020 Jul 14;14(7):e0008438. doi: 10.1371/journal.pntd.0008438 (PMC7380650; doi:10.1371/journal.pntd.0008438)
Supplement: S1 Table — (DOCX) [file pntd.0008438.s001.docx]

Supplementary materials

**S1 Table: Description of parameters used in *R_hv_ and R_hh_* calculation**

| **Parameter** | **Description** | **Estimate/Equation/Range** | | | **Relationship** | **Reference** |
| --- | --- | --- | --- | --- | --- | --- |
| $\boldsymbol{b}$ | Average daily vector biting rate (number of bites per day) | *Ae. aegypti* | 0.0043 x AvgT + 0.0943 | | Temperature and vector dependent | [87] |
|  |  | *Ae. albopictus* | 0.05 | |  | [88] |
|  |  |  | 0.16 | |  |  |
| $\boldsymbol{\beta}_{\boldsymbol{m}}$ | Probability of human to vector transmission per bite | *Ae. aegypti* | | 0.57 | Virus dependent | [37] |
|  |  |  |  | 0.83 |  | [27] |
|  |  |  |  | 0.70 |  | [35] |
|  |  | *Ae. albopictus* | | 0.73 |  | [27] |
|  |  |  |  | 0.90 |  | [35] |
| $\boldsymbol{\beta}_{\boldsymbol{h}}$ | Probability of vector to human transmission per bite | *Ae. aegypti* | | 0.27 |  | [37] |
|  |  |  |  | 0.87 |  | [27] |
|  |  |  |  | 0.55 |  | [35] |
|  |  | *Ae. albopictus* | | 0.76 |  | [27] |
|  |  |  |  | 0.10 |  | [35] |
| $\boldsymbol{\rho}$ | Relative human-to-mosquito transmission probability of exposed humans to symptomatically infected humans (per day) | 0.10 [0-0.30] | | |  | [56] |
| $\frac{\boldsymbol{1}}{\boldsymbol{\gamma}_{\boldsymbol{H}\boldsymbol{1}}}$ | Duration of acute phase | 5 [3-7] | | |  | [89] |
| $\frac{\boldsymbol{1}}{\boldsymbol{\gamma}_{\boldsymbol{H}}}$ | Intrinsic incubation period in humans | 5 [2-7] | | |  | [89] |
| $\frac{\boldsymbol{1}}{\boldsymbol{\gamma}_{\boldsymbol{v}}}$ | Duration of extrinsic incubation period (days) | *Ae. aegypti* | Lower | 7 |  | [26, 90-92] |
|  |  |  | Upper | 10 |  | [37, 93, 94] |
|  |  | *Ae. albopictus* | Lower | 10 |  | [30] |
|  |  |  | Upper | 14 |  | [26, 35] |
| $\boldsymbol{c}$ | Vector control rate (probability of survival) | Upper | | 0.1 | Species dependent | [95-97] |
|  |  | Lower | | 0.3 |  |  |
| $\boldsymbol{1/}\boldsymbol{\mu}_{\boldsymbol{v}}$ | Mosquito lifespan | 14 [4-35] | | |  | [93, 98] |
| $\boldsymbol{M}_{\boldsymbol{L}}$ | Mosquito population density in each location (Number host seeking females per hectare) | Calculated yearly using CIMSiM program. | | | Location dependent |  |
| $\boldsymbol{H}_{\boldsymbol{L}}$ | Human population density in each location (Number people at UCL level per hectare) | Calculated yearly using linear regression based on 1996, 2001, 2006, 2011 census data. | | |  | [50] |
| $\boldsymbol{K}$ | Relative human-to-human transmissibility of exposed humans to symptomatic humans. (dimensionless) | 0.6, [0-1] | | |  | [56] |
| $\boldsymbol{\beta}$ | Transmission rate from symptomatically infected humans to susceptible humans. (Per day) | 0.05, [0.001-0.10] | | |  | [56] |
| $\boldsymbol{\tau}$ | Relative human-to-human transmissibility of convalescent to symptomatic humans. (Dimensionless) | 0.3, [0-1] | | |  | [56] |
| $\boldsymbol{\theta}$ | Proportion of symptomatic infections. Dimensionless | 0.18, [0.10-0.27] | | |  | [20] |
| $\frac{\boldsymbol{1}}{\boldsymbol{\gamma}_{\boldsymbol{H}\boldsymbol{2}}}$ | Duration of convalescent phase. (Days) | 20, [14-30] | | |  | [89, 99, 100] |

ZIKV = Zika virus; AvgT = mean monthly temperature, CIMSiM = Container inhabiting mosquito simulation program; UCL = Urban Centres and Localities

1. Petersen LR, Busch MP. Transfusion-transmitted arboviruses. Vox Sanguinis. 2010;98(4):495-503. doi: 10.1111/j.1423-0410.2009.01286.x.

2. Hoad VC, Speers DJ, Keller AJ, Dowse GK, Seed CR, Lindsay MDA, et al. First reported case of transfusion-transmitted Ross River virus infection. Med J Aust. 2015;202(5):267-9. doi: 10.5694/mja14.01522.

3. Musso D, Nhan T, Robin E, Roche C, Bierlaire D, Zisou K, et al. Potential for Zika virus transmission through blood transfusion demonstrated during an outbreak in French Polynesia, November 2013 to February 2014. Euro Surveill 2014;19(14). doi: <http://dx.doi.org/10.2807/1560-7917.ES2014.19.14.20761>

4. Barjas-Castro ML, Angerami RN, Cunha MS, Suzuki A, Nogueira JS, Rocco IM, et al. Probable transfusion-transmitted Zika virus in Brazil. Transfusion. 2016;56(7):1684-8. Epub 2016/06/23. doi: 10.1111/trf.13681. PubMed PMID: 27329551.

5. Motta IJF, Spencer BR, Cordeiro da Silva SG, Arruda MB, Dobbin JA, Gonzaga YBM, et al. Evidence for transmission of Zika Virus by platelet transfusion. New England Journal of Medicine. 2016;375(11):1101-3. doi: doi:10.1056/NEJMc1607262. PubMed PMID: 27532622.

6. Jimenez A, Shaz BH, Kessler D, Bloch EM. How do we manage blood donors and recipients after a positive Zika screening result? Transfusion. 2017;57(9):2077-83. Epub 2017/07/25. doi: 10.1111/trf.14252. PubMed PMID: 28734023.

7. Cao-Lormeau V-M, Blake A, Mons S, Lastère S, Roche C, Vanhomwegen J, et al. Guillain-Barré Syndrome outbreak associated with Zika virus infection in French Polynesia: a case-control study. The Lancet. 2016;387(10027):1531-9. doi: <https://doi.org/10.1016/S0140-6736(16)00562-6>.

8. Musso D, Broult J, Bierlaire D, Lanteri MC, Aubry M. Prevention of transfusion-transmitted Zika virus in French Polynesia, nucleic acid testing versus pathogen inactivation. ISBT Science Series. 2017;12(1):254-9. doi: 10.1111/voxs.12335.

9. Paz-Bailey G, Rosenberg ES, Doyle K, Munoz-Jordan J, Santiago GA, Klein L, et al. Persistence of Zika Virus in body fluids - Preliminary report. N Engl J Med. 2017. Epub 2017/02/15. doi: 10.1056/NEJMoa1613108. PubMed PMID: 28195756.

10. Mead PS, Duggal NK, Hook SA, Delorey M, Fischer M, Olzenak McGuire D, et al. Zika Virus Shedding in Semen of Symptomatic Infected Men. N Engl J Med. 2018;378(15):1377-85. doi: 10.1056/NEJMoa1711038. PubMed PMID: 29641964.

11. Stone M, Bakkour S, Tzong-Hae L, Lanteri M, Simmons G, Brambilla D, et al. Zika RNA persistence in blood and body fluids and clinical outcomes in infected blood donors. Transfusion. 2017;57(3):4A.

12. Viennet E, Ritchie S, Faddy H, Williams C, Harley D. Epidemiology of dengue in a high-income country: a case study in Queensland, Australia. Parasites & Vectors. 2014;7(1):379. doi: 10.1186/1756-3305-7-379.

13. Beebe NW, Cooper RD, Mottram P, Sweeney AW. Australia's dengue risk driven by human adaptation to climate change. PLoS Neglected Tropical Diseases. 2009;3(5):9. PubMed PMID: ISI:000267268000003.

14. Trewin BJ, Darbro JM, Jansen CC, Schellhorn NA, Zalucki MP, Hurst TP, et al. The elimination of the dengue vector, *Aedes aegypti,* from Brisbane, Australia: The role of surveillance, larval habitat removal and policy. PLoS Negl Trop Dis. 2017;11(8):e0005848. Epub 2017/08/29. doi: 10.1371/journal.pntd.0005848. PubMed PMID: 28846682.

15. Beebe NW, Ambrose L, Hill LA, Davis JB, Hapgood G, Cooper RD, et al. Tracing the tiger: Population genetics provides valuable insights into the *Aedes* (*Stegomyia*) *albopictus.* Invasion of the Australasian region. PLoS Neglected Tropical Diseases. 2013;7(8):e2361. doi: 10.1371/journal.pntd.0002361.

16. Australian Government, Department of Agriculture and Water Resources. Media Statement: Managing exotic mosquitoes at the border 2016 [updated 04/02/201630/04/201]. Available from: <http://www.agriculture.gov.au/about/media-centre/media-releases/managing-exotic-mosquitoes-at-the-border>.

17. D’Ortenzio E, Matheron S, de Lamballerie X, Hubert B, Piorkowski G, Maquart M, et al. Evidence of sexual transmission of Zika virus. N Engl J Med. 2016;374(22):2195-8. doi: doi:10.1056/NEJMc1604449. PubMed PMID: 27074370.

18. Calvet G, Aguiar RS, Melo ASO, Sampaio SA, de Filippis I, Fabri A, et al. Detection and sequencing of Zika virus from amniotic fluid of fetuses with microcephaly in Brazil: a case study. Lancet Infect Dis. 2016;16(6):653-60. doi: 10.1016/S1473-3099(16)00095-5.

19. Besnard M, Lastere S, Teissier A, Cao-Lormeau V, Musso D. Evidence of perinatal transmission of Zika virus, French Polynesia, December 2013 and February 2014. Eurosurveillance. 2014;19(13):pii=20751.

20. Duffy MR, Chen T-H, Hancock WT, Powers AM, Kool JL, Lanciotti RS, et al. Zika virus outbreak on Yap Island, Federated States of Micronesia. New England Journal of Medicine. 2009;360(24):2536-43. doi: doi:10.1056/NEJMoa0805715. PubMed PMID: 19516034.

21. Aubry M, Teissier A, Huart M, Merceron S, Vanhomwegen J, Roche C, et al. Zika Virus seroprevalence, French Polynesia, 2014–2015. Emerging Infectious Disease journal. 2017;23(4). doi: 10.3201/eid2304.161549.

22. Mlakar J, Korva M, Tul N, Popović M, Poljšak-Prijatelj M, Mraz J, et al. Zika Virus associated with microcephaly. New England Journal of Medicine. 2016;374(10):951-8. doi: doi:10.1056/NEJMoa1600651. PubMed PMID: 26862926.

23. Parra B, Lizarazo J, Jiménez-Arango JA, Zea-Vera AF, González-Manrique G, Vargas J, et al. Guillain–Barré syndrome associated with Zika Virus infection in Colombia. New England Journal of Medicine. 2016;375(16):1513-23. doi: doi:10.1056/NEJMoa1605564. PubMed PMID: 27705091.

24. Countries and territories with current or previous Zika virus transmission [Internet]. 2 July 2019 [cited 27/09/2019]. Available from: <https://www.who.int/emergencies/diseases/zika/countries-with-zika-and-vectors-table.pdf>.

25. European Centre for Disease Prevention and Control. Zika virus transmission worldwide. Stockholm: ECDC, 9 April 2019.

26. Chouin-Carneiro T, Vega-Rua A, Vazeille M, Yebakima A, Girod R, Goindin D, et al. Differential susceptibilities of *Aedes aegypti* and *Aedes albopictus* from the Americas to Zika virus. PLoS Negl Trop Dis. 2016;10(3):e0004543. doi: 10.1371/journal.pntd.0004543.

27. Duchemin J-B, Mee PT, Lynch SE, Vedururu R, Trinidad L, Paradkar P. Zika vector transmission risk in temperate Australia: a vector competence study. Virology Journal. 2017;14(1):108. doi: 10.1186/s12985-017-0772-y.

28. Jupille H, Seixas G, Mousson L, Sousa CA, Failloux A-B. Zika Virus, a new threat for Europe? PLoS Negl Trop Dis. 2016;10(8):e0004901. doi: 10.1371/journal.pntd.0004901.

29. Zhuanzhuan L, Tengfei Z, Zetian L, Zhenhong Z, Zhirong J, Guofa Z, et al. Competence of *Aedes aegypti*, *Ae. albopictus*, and *Culex quinquefasciatus* mosquitoes as Zika virus vectors, China. Emerging Infectious Disease journal. 2017;23(7):1085. doi: 10.3201/eid2307.161528.

30. Wong P-SJ, Li M-zI, Chong C-S, Ng L-C, Tan C-H. *Aedes* (Stegomyia) *albopictus* (Skuse): A potential vector of Zika virus in Singapore. PLOS Neglected Tropical Diseases. 2013;7(8):e2348. doi: 10.1371/journal.pntd.0002348.

31. Ryckebusch F, Berthet M, Missé D, Choumet V. Infection of a French population of *Aedes albopictus* and of *Aedes aegypti* (Paea Strain) with Zika virus reveals low transmission rates to these vectors’ saliva. International Journal of Molecular Sciences. 2017;18(11):2384. PubMed PMID: doi:10.3390/ijms18112384.

32. Azar SR, Roundy CM, Rossi SL, Huang JH, Leal G, Yun R, et al. Differential vector competency of *Aedes albopictus* populations from the Americas for Zika virus. The American Journal of Tropical Medicine and Hygiene. 2017;97(2):330-9. doi: <https://doi.org/10.4269/ajtmh.16-0969>.

33. Di Luca M, Severini F, Toma L, Boccolini D, Romi R, Remoli ME, et al. Experimental studies of susceptibility of Italian <italic>Aedes albopictus</italic> to Zika virus. Eurosurveillance. 2016;21(18). doi: 16-00296. PubMed PMID: SC000015507.

34. Pompon J, Morales-Vargas R, Manuel M, Huat Tan C, Vial T, Hao Tan J, et al. A Zika virus from America is more efficiently transmitted than an Asian virus by *Aedes aegypti* mosquitoes from Asia. Sci Rep. 2017;7(1):1215. Epub 2017/04/30. doi: 10.1038/s41598-017-01282-6. PubMed PMID: 28450714; PubMed Central PMCID: PMCPMC5430906.

35. Hugo RLE, Stassen L, La J, Gosden E, Ekwudu O, Winterford C, et al. Vector competence of Australian *Aedes aegypti* and *Aedes albopictus* for an epidemic strain of Zika virus. PLoS Negl Trop Dis. 2019;13(4):e0007281. Epub 2019/04/05. doi: 10.1371/journal.pntd.0007281. PubMed PMID: 30946747.

36. Hall-Mendelin S, Pyke AT, Moore PR, Ritchie SA, Moore FAJ, van den Hurk AF. Characterization of a Western Pacific Zika Virus Strain in Australian Aedes aegypti. Vector-Borne and Zoonotic Diseases. 2018;18(6):317-22. doi: 10.1089/vbz.2017.2232.

37. Hall-Mendelin S, Pyke AT, Moore PR, Mackay IM, McMahon JL, Ritchie SA, et al. Assessment of local mosquito species incriminates *Aedes aegypti* as the potential vector of Zika Virus in Australia. PLoS Negl Trop Dis. 2016;10(9):e0004959. doi: 10.1371/journal.pntd.0004959.

38. NNDSS Annual Report Writing Group. Australia's notifiable disease status, 2012: Annual report of the National Notifiable Diseases Surveillance System. Commun Dis Intell. 2015;39(1).

39. NNDSS Annual Report Writing Group. Australia's notifiable disease status, 2013: Annual report of the national notifiable diseases surveillance system. Commun Dis Intell. 2015;39(3).

40. Department of Health, National Notifiable Diseases Surveillance System. Overseas - acquired vectorborne disease notifications in Australia - Fortnight ending 23 March 2019 2019.

41. Watson-Brown P, Viennet E, Hoad VC, Flower RLP, Faddy HM. Is Zika virus a potential threat to the Australian Blood Supply? Aust N Z J Public Health. 2017. Epub 2017/07/28. doi: 10.1111/1753-6405.12697. PubMed PMID: 28749569.

42. Watson-Brown P, Viennet E, Mincham G, Williams CR, Jansen CC, Montgomery BL, et al. Epidemic potential of Zika virus in Australia: implications for blood transfusion safety. Transfusion. 2019. Epub 2019/01/09. doi: 10.1111/trf.15095. PubMed PMID: 30618208.

43. Kamtchum-Tatuene J, Makepeace BL, Benjamin L, Baylis M, Solomon T. The potential role of *Wolbachia* in controlling the transmission of emerging human arboviral infections. Current opinion in infectious diseases. 2017;30(1):108-16. doi: 10.1097/QCO.0000000000000342. PubMed PMID: 27849636.

44. Ritchie SA, van den Hurk AF, Smout MJ, Staunton KM, Hoffmann AA. Mission Accomplished? We Need a Guide to the 'Post Release' World of Wolbachia for Aedes-borne Disease Control. Trends Parasitol. 2018;34(3):217-26. Epub 2018/02/06. doi: 10.1016/j.pt.2017.11.011. PubMed PMID: 29396201.

45. Russell RC, Currie BJ, Lindsay MD, Mackenzie JS, Ritchie SA, Whelan PI. Dengue and climate change in Australia: predictions for the future should incorporate knowledge from the past. Medical Journal of Australia. 2009;190(5):265-8. PubMed PMID: ISI:000265400000015.

46. Australian Red Cross Blood Service. Zika virus update Transfusion News. 2016;(updated 12 Dec 2016).

47. Australian Red Cross Blood Service. Donor questionnaire. 2016.

48. Australian Red Cross Blood Service. Guidelines for the Selection of Blood Donors. Australia, 2014. 2014. Updated on 7 January, 2019.

49. Viennet E, Mincham G, Frentiu FD, Jansen CC, Montgomery BL, Harley D, et al. Epidemic Potential for Local Transmission of Zika Virus in 2015 and 2016 in Queensland, Australia. PLoS Curr. 2016;8. Epub 2017/01/27. doi: 10.1371/currents.outbreaks.73d82b08998c6d729c41ef6cdcc80176. PubMed PMID: 28123859; PubMed Central PMCID: PMCPMC5222544.

50. Australian Bureau of Statistics. Census Community Profiles by location 2012. Available from: <http://www.abs.gov.au/websitedbs/censushome.nsf/home/communityprofiles?opendocument&navpos=230>.

51. Muzari MO, Davis J, Bellwood R, Crunkhorn B, Gunn E, Sabatino U, et al. Dominance of the tiger: The displacement of *Aedes aegypti* by *Aedes albopictus* in parts of the Torres Strait, Australia. Commun Dis Intell. 2019. doi: 10.33321/cdi.2019.43.17.

52. Climate data online [Internet]. 2016 [cited 14 July 2016]. Available from: <http://www.bom.gov.au/climate/data/index.shtml?bookmark=200>.

53. Focks D, Haile D, Daniels E, Mount G. Dynamic life table model for Ae aegypti (Diptera:Culicidae): analysis of the literature and model development. J Med Entomol. 1993;30:1003 - 17.

54. Williams C, Johnson PH, Long SA, Rapley LP, Ritchie SA. Rapid estimation of *Aedes aegypti* population size using simulation modeling, with a novel approach to calibration and field validation. Journal of Medical Entomology. 2008;45(6):1173-9.

55. Villela DAM, Bastos LS, De Carvalho LM, Cruz OG, Gomes MFC, Durovni B, et al. Zika in Rio de Janeiro: Assessment of basic reproduction number and comparison with dengue outbreaks. Epidemiology and Infection. 2017;145(8):1649-57. Epub 02/27. doi: 10.1017/S0950268817000358.

56. Gao D, Lou Y, He D, Porco TC, Kuang Y, Chowell G, et al. Prevention and control of Zika as a mosquito-borne and sexually transmitted disease: A mathematical modeling analysis. Scientific Reports. 2016;6:28070. doi: 10.1038/srep28070.

57. Anderson R, May R. Infectious diseases of humans: dynamics and control. 1991.

58. R Core Team. R: A language and environment for statistical computing. R Foundation for Statistical Computing. 2017.

59. Marino S, Hogue IB, Ray CJ, Kirschner DE. A methodology for performing global uncertainty and sensitivity analysis in systems biology. Journal of Theoretical Biology. 2008;254(1):178-96. doi: <https://doi.org/10.1016/j.jtbi.2008.04.011>.

60. Crosetto M, Tarantola S, Saltelli A. Sensitivity and uncertainty analysis in spatial modelling based on GIS. Agriculture, Ecosystems and Environment. 2000;81:71 - 9. PubMed PMID: doi:10.1016/S0167-8809(00)00169-9.

61. Keeling M, Rohani P. Modeling infectious diseases in humans and animals. Press PU, editor. Princeton2007.

62. Perkins TA, Siraj AS, Ruktanonchai CW, Kraemer MUG, Tatem AJ. Model-based projections of Zika virus infections in childbearing women in the Americas. Nature Microbiology. 2016;1:16126. doi: 10.1038/nmicrobiol.2016.126

<https://www.nature.com/articles/nmicrobiol2016126#supplementary-information>.

63. Seed CR, Hoad VC, Faddy HM, Kiely P, Keller AJ, Pink J. Re-evaluating the residual risk of transfusion-transmitted Ross River virus infection. Vox Sang. 2016;110(4):317-23. Epub 2016/01/11. doi: 10.1111/vox.12372. PubMed PMID: 26748600.

64. Oei W, Janssen MP, van der Poel CL, van Steenbergen JE, Rehmet S, Kretzschmar ME. Modeling the transmission risk of emerging infectious diseases through blood transfusion. Transfusion. 2013;53(7):1421-8. Epub 2012/11/02. doi: 10.1111/j.1537-2995.2012.03941.x. PubMed PMID: 23113823.

65. Oei W, Lieshout-Krikke RW, Kretzschmar ME, Zaaijer HL, Coutinho RA, Eersel M, et al. Estimating the risk of dengue transmission from Dutch blood donors travelling to Suriname and the Dutch Caribbean. Vox Sang. 2016;110(4):301-9. Epub 2016/01/15. doi: 10.1111/vox.12370. PubMed PMID: 26765798.

66. Coghlan A, Hoad VC, Seed CR, Flower RL, Harley RJ, Herbert D, et al. Emerging infectious disease outbreaks: estimating disease risk in Australian blood donors travelling overseas. Vox Sang. 2017. Epub 2017/10/21. doi: 10.1111/vox.12571. PubMed PMID: 29052242.

67. Janssen MP. EUFRAT User Manual. 2015.

68. Kiely P, Gambhir M, Cheng AC, McQuilten ZK, Seed CR, Wood EM. Emerging Infectious Diseases and Blood Safety: Modeling the Transfusion-Transmission Risk. Transfusion Medicine Reviews. 2017;31(3):154-64. doi: 10.1016/j.tmrv.2017.05.002.

69. 1270.0.55.001 - Australian Statistical Geography Standard (ASGS): Volume 1 - Main Structure and Greater Capital City Statistical Areas, July 2016 [Internet]. 2016. Available from: <http://www.abs.gov.au/ausstats/abs@.nsf/Lookup/by%20Subject/1270.0.55.001~July%202016~Main%20Features~Statistical%20Area%20Level%203%20(SA3)~10015>.

70. 1270.0.55.001 - Australian Statistical Geography Standard (ASGS): Volume 1 - Main Structure and Greater Capital City Statistical Areas, July 2016 [Internet]. 2016. Available from: <http://www.abs.gov.au/ausstats/abs@.nsf/Lookup/by%20Subject/1270.0.55.001~July%202016~Main%20Features~Statistical%20Area%20Level%201%20(SA1)~10013>.

71. Australian Bureau of Statistics. Australian Statistical Geography Standard (ASGS): Volume 4 - Significant Urban Areas, Urban Centres and Localities, Section of State. July 2011.

72. Krzywinski M, Altman N. Visualizing samples with box plots. Nature methods. 2014;11(2):119-20. Epub 2014/03/20. PubMed PMID: 24645192.

73. Kraemer MUG, Sinka ME, Duda KA, Mylne AQN, Shearer FM, Barker CM, et al. The global distribution of the arbovirus vectors *Aedes aegypti* and *Ae. albopictus*. eLife. 2015;4:e08347. doi: 10.7554/eLife.08347.

74. Kucharski AJ, Funk S, Eggo RM, Mallet H-P, Edmunds WJ, Nilles EJ. Transmission dynamics of Zika Virus in Island populations: A modelling analysis of the 2013–14 French Polynesia outbreak. PLoS Negl Trop Dis. 2016;10(5):e0004726. doi: 10.1371/journal.pntd.0004726.

75. Rahman M, Bekele-Maxwell K, Cates LL, Banks HT, Vaidya NK. Modeling Zika Virus Transmission Dynamics: Parameter Estimates, Disease Characteristics, and Prevention. Scientific Reports. 2019;9(1):10575. doi: 10.1038/s41598-019-46218-4.

76. World Health Organization. Prevention of sexual transmission of Zika virus. 2016.

77. Germain M, Delage G, O'Brien SF, Grégoire Y, Fearon M, Devine D. Mitigation of the threat posed to transfusion by donors traveling to Zika‐affected areas: a Canadian risk‐based approach. Transfusion. 2017;57(10):2463-8. doi: 10.1111/trf.14247.

78. Lazear HM, Diamond MS. Zika virus: New clinical syndromes and its emergence in the Western Hemisphere. J Virol. 2016;90(10):4864-75. Epub 2016/03/11. doi: 10.1128/jvi.00252-16. PubMed PMID: 26962217; PubMed Central PMCID: PMCPMC4859708.

79. Flamand C, Fritzell C, Matheus S, Dueymes M, Carles G, Favre A, et al. The proportion of asymptomatic infections and spectrum of disease among pregnant women infected by Zika virus: systematic monitoring in French Guiana, 2016. Eurosurveillance. 2017;22(44):17-00102. doi: 10.2807/1560-7917.ES.2017.22.44.17-00102. PubMed PMID: PMC5710134.

80. Joguet G, Mansuy J-M, Matusali G, Hamdi S, Walschaerts M, Pavili L, et al. Effect of acute Zika virus infection on sperm and virus clearance in body fluids: a prospective observational study. The Lancet Infectious Diseases. 2017;17(11):1200-8. doi: <https://doi.org/10.1016/S1473-3099(17)30444-9>.

81. Stassen L, Armitage CW, Van der Heide DJ, Beagley KW, Frentiu FD. Zika virus in the male reproductive tract. Viruses. 2018;10(4):198. PubMed PMID: doi:10.3390/v10040198.

82. Aliota MT, Peinado SA, Velez ID, Osorio JE. The wMel strain of *Wolbachia* reduces transmission of Zika virus by *Aedes aegypti*. Sci Rep. 2016;6:28792. Epub 2016/07/02. doi: 10.1038/srep28792. PubMed PMID: 27364935; PubMed Central PMCID: PMCPMC4929456.

83. Maharajan MK, Ranjan A, Chu JF, Foo WL, Chai ZX, Lau EY, et al. Zika virus infection: current concerns and perspectives. Clin Rev Allergy Immunol. 2016;51(3):383-94. Epub 2016/05/30. doi: 10.1007/s12016-016-8554-7. PubMed PMID: 27236440.

84. Dodd RY. Getting to know the unknown. Transfusion. 2014;54(7):1693-4. doi: 10.1111/trf.12705.

85. Devine DV, Schubert P. Pathogen Inactivation Technologies: The Advent of Pathogen-Reduced Blood Components to Reduce Blood Safety Risk. Hematology/Oncology Clinics. 2016;30(3):609-17. doi: 10.1016/j.hoc.2016.01.005.

86. McCullough J. Pathogen inactivation: a new paradigm for preventing transfusion-transmitted infections. Am J Clin Pathol. 2007;128(6):945-55. Epub 2007/11/21. doi: 10.1309/rapq3nxg3mv9al94. PubMed PMID: 18024320.

87. Liu-Helmersson J, Stenlund H, Wilder-Smith A, Rocklöv J. Vectorial capacity of *Aedes aegypti*: Effects of temperature and implications for global dengue epidemic potential. PLoS ONE. 2014;9(3):e89783. doi: 10.1371/journal.pone.0089783.

88. Poletti P, Messeri G, Ajelli M, Vallorani R, Rizzo C, Merler S. Transmission potential of Chikungunya virus and control measures: The case of Italy. Plos One. 2011;6:e18860.

89. Bearcroft WGC. Zika virus infection experimentally induced in a human volunteer. Trans R Soc Trop Med Hyg. 1956;50(5):442-8.

90. Li MI, Wong PSJ, Ng LC, Tan CH. Oral Susceptibility of Singapore *Aedes* (*Stegomyia*) *aegypti* (Linnaeus) to Zika Virus. PLoS Negl Trop Dis. 2012;6(8):e1792. doi: 10.1371/journal.pntd.0001792.

91. Rocklöv J, Quam MB, Sudre B, German M, Kraemer MUG, Brady O, et al. Assessing Seasonal Risks for the Introduction and Mosquito-borne Spread of Zika Virus in Europe. EBioMedicine. 2016. doi: <http://dx.doi.org/10.1016/j.ebiom.2016.06.009>.

92. Wong PS, Li MZ, Chong CS, Ng LC, Tan CH. *Aedes (Stegomyia) albopictus* (Skuse): a potential vector of Zika virus in Singapore. PLoS Negl Trop Dis. 2013;7(8):e2348. Epub 2013/08/13. doi: 10.1371/journal.pntd.0002348. PubMed PMID: 23936579; PubMed Central PMCID: PMCPMC3731215.

93. Boorman JP, Porterfield JS. A simple technique for infection of mosquitoes with viruses; transmission of Zika virus. Trans R Soc Trop Med Hyg. 1956;50(3):238-42. Epub 1956/05/01. PubMed PMID: 13337908.

94. Rojas DP, Dean NE, Yang Y, Kenah E, Quintero J, Tomasi S, et al. The epidemiology and transmissibility of Zika virus in Girardot and San Andres island, Colombia, September 2015 to January 2016. Euro Surveill. 2016;21(28). Epub 2016/07/28. doi: 10.2807/1560-7917.es.2016.21.28.30283. PubMed PMID: 27452806.

95. Hanna JN, Ritchie SA, Phillips DA, Serafin IL, Hills SL, van den Hurk AF, et al. An epidemic of dengue 3 in far north Queensland, 1997-1999. Medical Journal of Australia. 2001;174(4):178-82. PubMed PMID: ISI:000167135900007.

96. Muzari MO, Devine G, Davis J, Crunkhorn B, van den Hurk A, Whelan P, et al. Holding back the tiger: Successful control program protects Australia from *Aedes albopictus* expansion. PLoS Neglected Tropical Diseases. 2017;11(2):e0005286. doi: 10.1371/journal.pntd.0005286. PubMed PMID: PMC5305203.

97. Vazquez-Prokopec GM, Montgomery BL, Horne P, Clennon JA, Ritchie SA. Combining contact tracing with targeted indoor residual spraying significantly reduces dengue transmission. Science Advances. 2017;3(2):e1602024. doi: 10.1126/sciadv.1602024. PubMed PMID: PMC5315446.

98. Andraud M, Hens N, Marais C, Beutels P. Dynamic Epidemiological Models for Dengue Transmission: A Systematic Review of Structural Approaches. Plos One. 2012;7(11):e49085. doi: 10.1371/journal.pone.0049085.

99. Gourinat AC, O'Connor O, Calvez E, Goarant C, Dupont-Rouzeyrol M. Detection of Zika virus in urine. Emerg Infect Dis. 2015;21(1):84-6. Epub 2014/12/23. doi: 10.3201/eid2101.140894. PubMed PMID: 25530324; PubMed Central PMCID: PMCPMC4285245.

100. Musso D, Roche C, Robin E, Nhan T, Teissier A, Cao-Lormeau V-M. Potential Sexual Transmission of Zika Virus. Emerging Infectious Diseases. 2015;21(2):359-61. doi: 10.3201/eid2102.141363. PubMed PMID: PMC4313657.
