# Supplementary figures and images for: Estimation of mosquito-borne and sexual transmission of Zika virus in Australia: Risks to blood transfusion safety
Source: PLoS Negl Trop Dis. 2020 Jul 14;14(7):e0008438. doi: 10.1371/journal.pntd.0008438 (PMC7380650; doi:10.1371/journal.pntd.0008438)

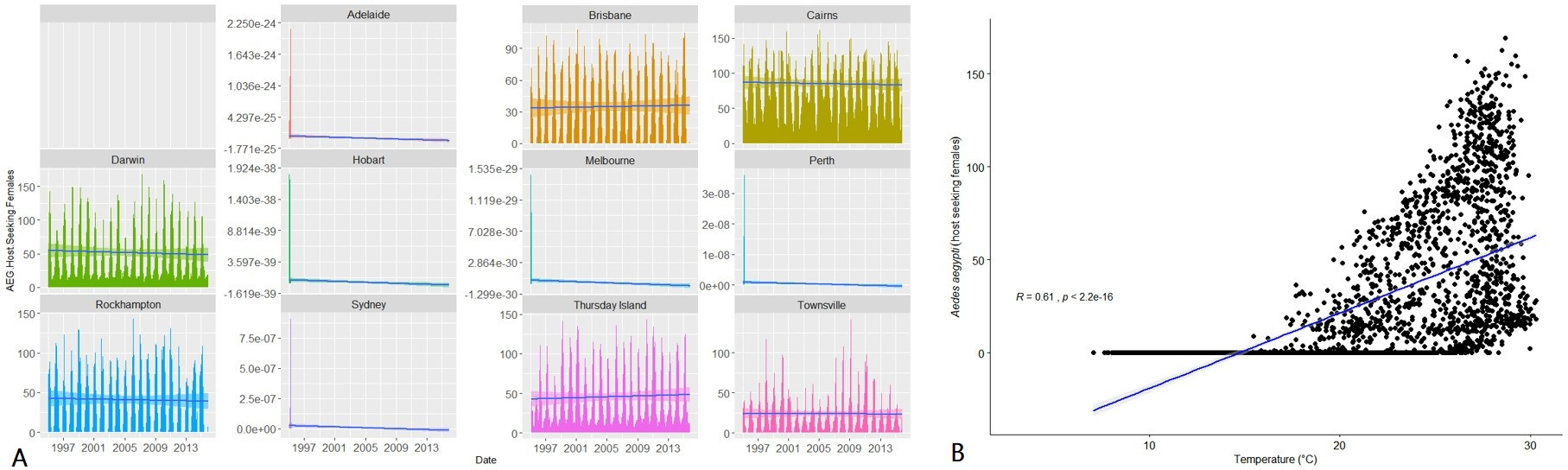

Supplement: S1 Fig — (A) Distribution from CIMSiM estimations through study period; (B) Analysis of correlation between the density of population and the Temperature (⁰C). (TIF) [file pntd.0008438.s002.tif]

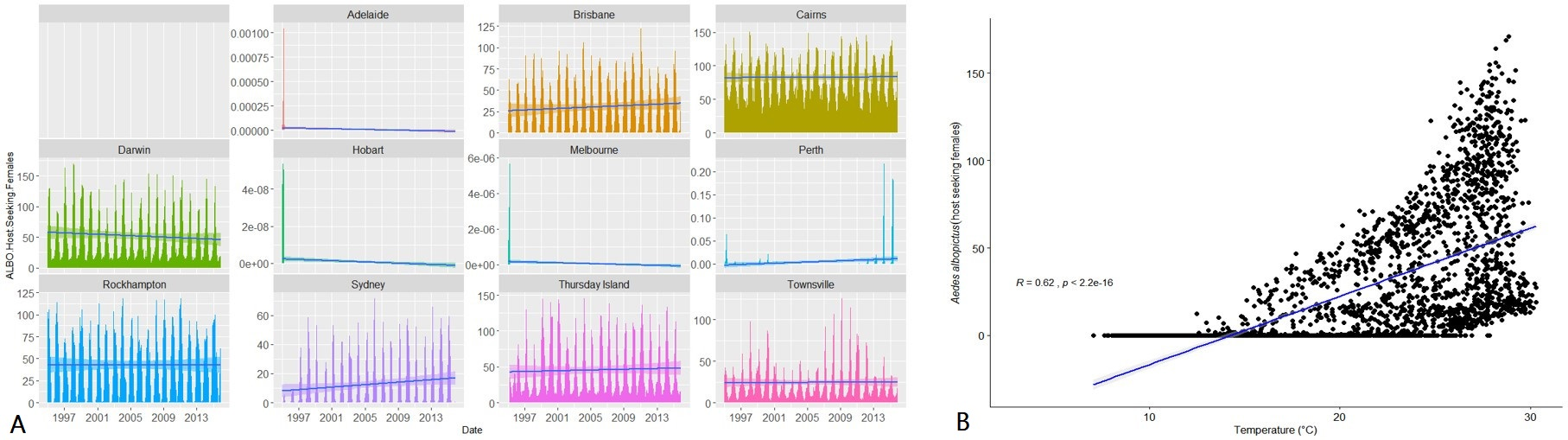

Supplement: S2 Fig — (A) Distribution from CIMSiM estimations through study period; (B) Analysis of correlation between the density of population and the Temperature (⁰C). (TIF) [file pntd.0008438.s003.tif]

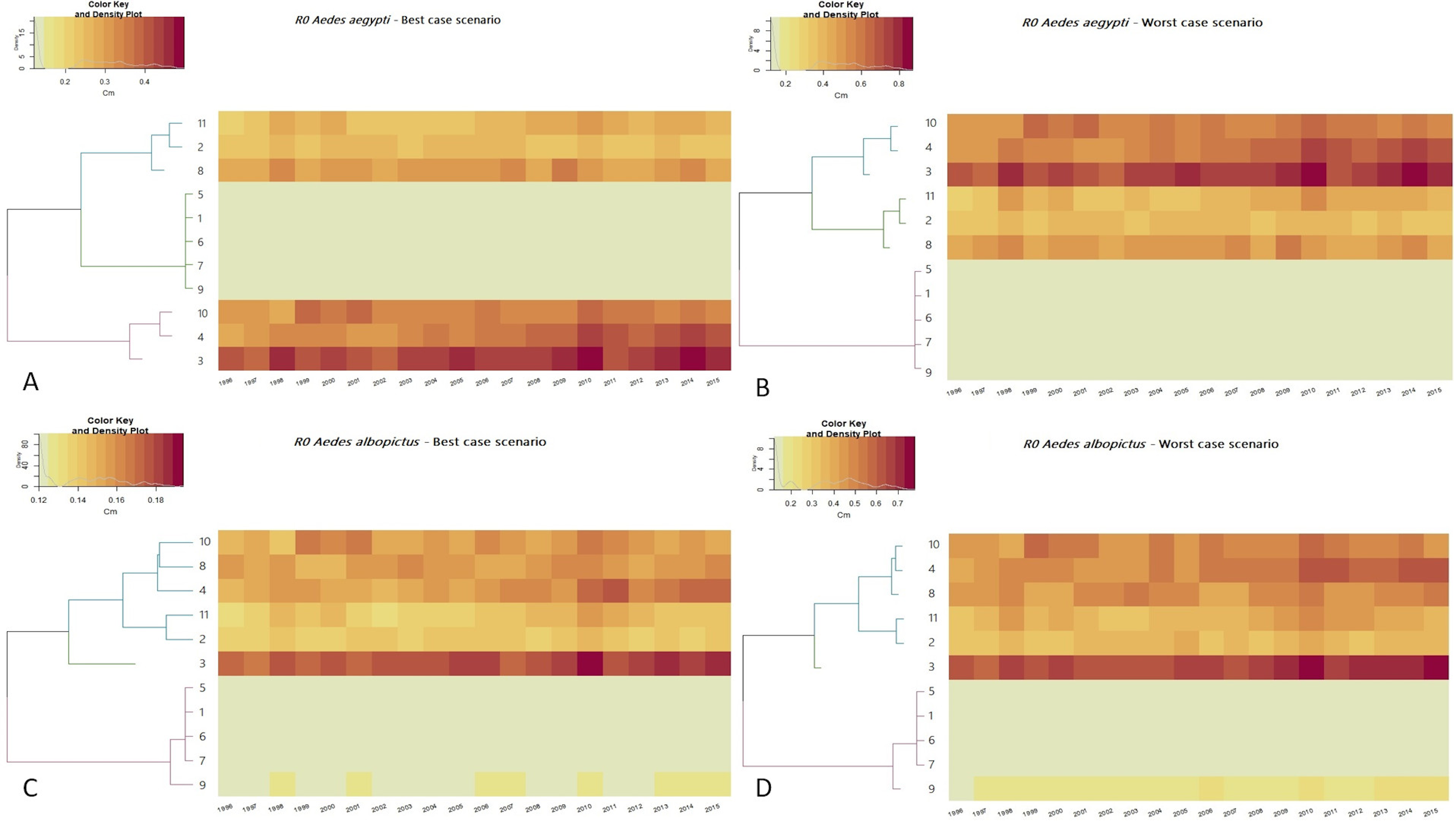

Supplement: S3 Fig — Heat map of R0 by Urban Centres and Localities throughout from 2009–2015. (A) ‘best-‘case scenario with Aedes aegypti; (B) ‘worst-‘case scenario with Ae. aegypti; (C) ‘best-‘case scenario with Aedes albopictus; (D) ‘worst-‘case scenario with Ae. Albopictus 1.Adelaide; 2. Brisbane; 3. Cairns; 4. Darwin; 5. Hobart; 6. Melbourne; 7. Perth; 8. Rockhampton; 9. Sydney; 10. Thursday Island; 11. Townsville. (TIF) [file pntd.0008438.s004.tif]

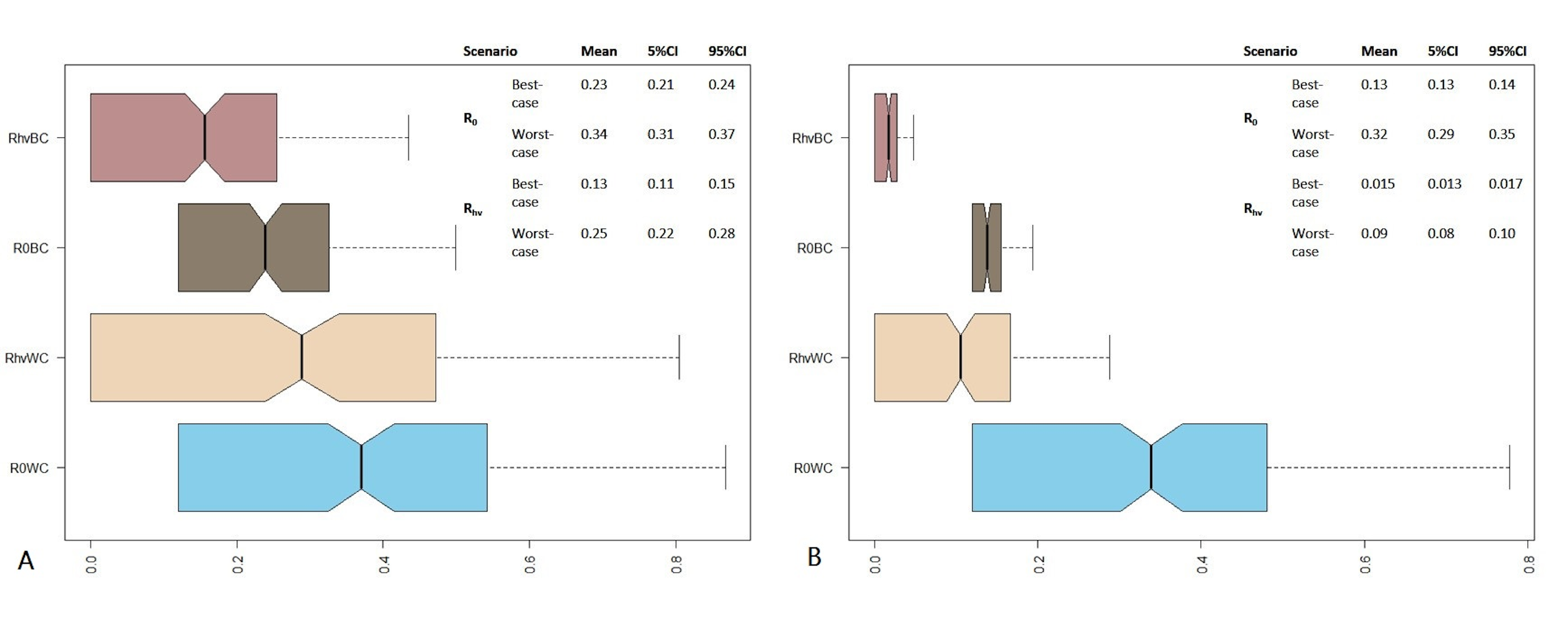

Supplement: S4 Fig — (A) Notched Box plots for Aedes aegypti; (B) Notched Box plots for Aedes albopictus. The box shows the interquartile range. The whiskers add 1.5 times the IQR to the 75 percentiles and subtract 1.5 times the IQR from the 25 percentiles. The line shows the median of the data. The notch displays the confidence interval around the median which is normally based on the median. (TIF) [file pntd.0008438.s005.tif]

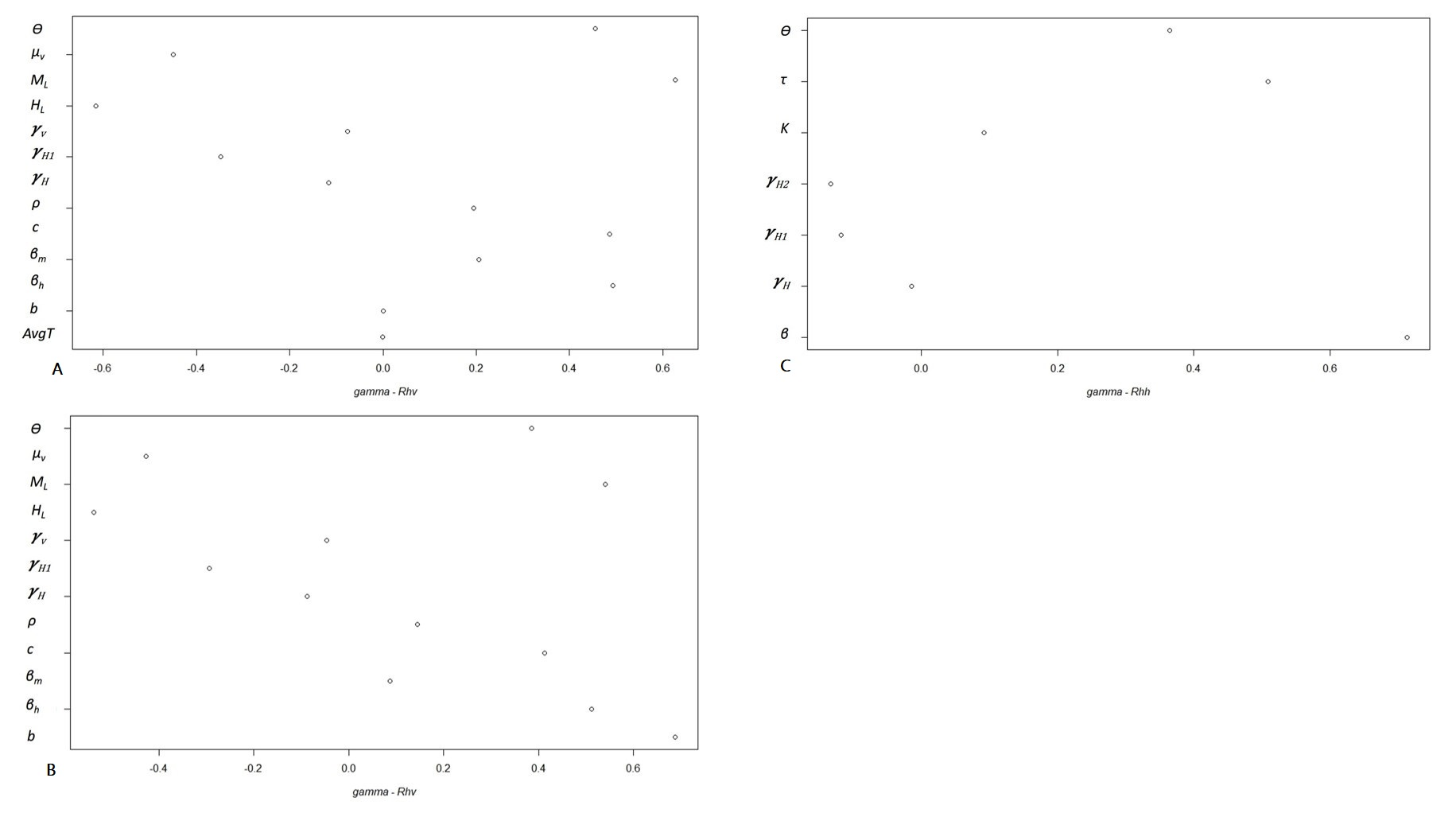

Supplement: S5 Fig — (A) via mosquito transmission and Aedes aegypti; (B) via mosquito transmission and Aedes albopictus;(C) via sexual transmission. (TIF) [file pntd.0008438.s006.tif]
